# Small Nucleolar RNAs: Biological Functions and Diseases

**DOI:** 10.1002/mco2.70257

**Published:** 2025-06-27

**Authors:** Yi Wang, Min Fu, Zaiyong Zheng, Jianguo Feng, Chunxiang Zhang

**Affiliations:** ^1^ Department of Anesthesiology The Affiliated Hospital Southwest Medical University Luzhou Sichuan Province China; ^2^ Anesthesiology and Critical Care Medicine Key Laboratory of Luzhou The Affiliated Hospital Southwest Medical University Luzhou Sichuan Province China; ^3^ School of Basic Medical Science Southwest Medical University Luzhou Sichuan China; ^4^ Department of Cardiology The Affiliated Hospital Key Laboratory of Medical Electrophysiology Ministry of Education Institute of Cardiovascular Research Basic Medicine Research Innovation Center for Cardiometabolic Diseases Ministry of Education Southwest Medical University Luzhou Sichuan China

**Keywords:** cancer, cardiovascular diseases, nucleic acid‐based drugs, neurological diseases, small nucleolar RNAs

## Abstract

Small nucleolar RNAs (snoRNAs) are a class of small RNA molecules that play a pivotal role in diverse cellular processes and are extensively implicated in the pathophysiology of various diseases. Here, we provide a comprehensive overview of snoRNAs, encompassing their classification, biogenesis, and both canonical and noncanonical functions. Canonical roles include guiding RNA modifications such as 2′‐O‐methylation, pseudouridylation, and N4‐acetylcytidine modification in targeted RNA molecules, as well as facilitating ribosome biogenesis. Noncanonical roles involve regulating mRNA processing, modulating alternative splicing, generating snoRNA‐derived small RNAs, and interacting with long noncoding RNAs. Dysregulation of snoRNAs has been implicated in various human diseases, such as cancer, neurodegenerative disorders, cardiovascular diseases, immunity‐ and inflammation‐related conditions, and aging, highlighting their potential as diagnostic and prognostic biomarkers. Advances in high‐throughput sequencing, structural biology, and bioinformatics tools have significantly contributed to the detection, screening, and exploration of the intricate biofunctions of snoRNAs. However, despite these technological advancements, challenges remain in unraveling the biological complexity of snoRNAs and translating these findings into clinical applications. This review discusses the current state of snoRNA research, recent technological breakthroughs, and future directions, emphasizing their emerging roles in health and disease.

## Graphical abstract

The majority of snoRNAs are derived from the intron region of genes, besides to intronic snoRNAs, some snoRNAs are transcribed as independent genes, often by Pol II or III, these snoRNAs are called intergenic snoRNAs. All of these play a role in the occurrence and progression of various diseases through multiple functions. Figures were created with biorender.com.

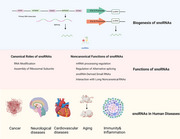

## 1 Introduction

Research into small nucleolar RNAs (snoRNAs) began in the late 1960s [1]. Over the decades, it became evident that snoRNAs, noncoding RNAs (ncRNAs) ranging from 60 to 300 nucleotides (nt), are localized within the nucleolus [2, 3]. Based on their structural characteristics, snoRNAs can be classified into two main types: C/D box and H/ACA box snoRNAs [4]. snoRNAs play crucial roles in pre‐ribosomal RNA (pre‐rRNA) modification and ribosomal RNA (rRNA) processing. Previous studies have found that snoRNAs not only participate in the modification of pre‐rRNA and processing of rRNA, but also regulate the function of ribosomes and spliceosomes by methylating and pseudouridylating nucleotide residues in ribosomal and small nuclear RNAs (snRNAs), respectively [5]. They also participate in posttranscriptional regulation through rRNA acetylation, modulation of splicing patterns, and control of mRNA abundance and translation efficiency [6]. Beyond their typical functions in the nucleolus, snoRNAs also play crucial roles in atypical mechanisms such as the biogenesis of PIWI‐interacting RNAs (piRNAs), snoRNA‐derived fragments (sdRNAs), certain microRNA (miRNA) derivatives, tRNA methylation, and rRNA acetylation. snoRNAs also play diverse roles in RNA‐related processes such as regulating splicing and chromatin accessibility [7]. It is well known that mRNA 3′ processing has a decisive impact on transcription turnover, translation efficiency, and cellular mRNA transport [8]. More importantly, research has found that the vast majority of RNAs associated with mRNA 3′ processing are snoRNAs [9]. These findings suggested that snoRNAs directly affect mRNA transcription, translation, and transport. Epigenetic processes associated with snoRNAs play crucial roles in the proper execution of cellular biological activities.

Current research on the role of snoRNAs in various diseases is rapidly growing. Alterations in the expression profile of snoRNAs have been reported in various diseases, including malignancies, infections, and other pathological conditions [10]. For instance, snoRNAs play significant roles in tumor diagnosis and prognosis, early detection of neurological and cardiovascular diseases (CVDs), and regulation of the pathogenesis of diseases related to the hematologic system, genetic disorders, immune inflammation, and so on. Therefore, snoRNAs have profound implications in cellular processes and disease pathogenesis, making them potential targets for therapeutic interventions and as diagnostic markers.

In this review, we elucidate the diverse biological functions of snoRNAs encompassing regulation of 2′‐O‐methylation, pseudouridylation, and N4‐acetylcytidine (ac4C) modification in targeted RNA molecules, as well as modulation of mRNA maturation, translation, stability, and alternative splicing process in genes. Furthermore, we provide a comprehensive summary on the implications of snoRNAs in various diseases such as cancers, neurological diseases, CVDs, inflammation, and aging. A deep understanding of the complex biological functions of snoRNAs and their involvement in these pathological conditions will greatly contribute to improving the accuracy of diagnosis, efficacy of treatment, and prediction of prognosis for related diseases.

## 2 Classification and Biogenesis of snoRNAs

### 2.1 Classification of snoRNAs

SnoRNAs are predominantly located in the nucleolus, a substructure of the nucleus where ribosomal biogenesis occurs. This localization is closely related to its role in rRNA processing [11, 12, 13]. The transcription of snoRNAs occurs through several mechanisms: (1) Independent transcription units: In intergenic regions, snoRNAs can exist as independent transcription units, either encoding a single snoRNA or forming intergenic snoRNA clusters with other snoRNAs [14, 15, 16, 17]. (2) Embedded within host genes: snoRNAs can also be embedded within host genes, typically located in the introns of the host gene. Their expression depends on host gene transcription. A single intronic snoRNA exists alone within an intron, whereas intronic snoRNA clusters refer to multiple snoRNAs present within the same intron [18, 19, 20, 21]. (3) Overlapping exonic sequences: Some snoRNA genes overlap with the exonic sequences of host genes. They can exist independently or form exonic snoRNA clusters, although exonic snoRNAs are relatively rare [22, 23, 24, 25, 26]. Based on their characteristic nucleotide motifs and protein‐binding properties, snoRNAs are classified into two subfamilies: C/D‐box and H/ACA‐box [2, 5, 27]. C/D box snoRNAs primarily facilitate 2′‐O‐ribose methylation processes. In contrast, H/ACA box snoRNAs guide pseudouridylation events [28, 29]. These structural motifs are essential for their function and for the assembly of snoRNA ribonucleoprotein (snoRNP) complexes [11, 12]. There is a unique subfamily of snoRNAs known as small Cajal body‐specific RNAs (scaRNAs). Both snoRNAs and scaRNAs share common features, with scaRNAs containing additional conserved sequence motifs, such as the CAB box (5′‐UGAC‐3′) in H/ACA Small Cajal body‐specific ribonucleoproteins (scaRNPs) and a GU/UG wobble stem in C/D scaRNPs [30, 31]. These motifs help localize these complexes to the Cajal body [32]. Interestingly, some snoRNAs, devoid of identified targets, are classified as orphan snoRNAs, and their biological functions remain largely unexplored.

### 2.2 Biogenesis of snoRNAs

Most snoRNAs are distributed in the introns of protein‐coding genes or long noncoding RNAs (lncRNAs) and are transcribed by RNA polymerase II (Pol II) [18, 33]. These snoRNAs, referred to as intronic snoRNAs, are processed from their host transcripts. Intronic snoRNAs are generated from host transcripts by splicing via the spliceosome and are then debranched by debranching enzyme 1. Subsequently, the pre‐snoRNAs are trimmed by exonucleases XRN2/XRN1 at the 5′ site and the nuclear exosome at the 3′ site [34]. Given that alternative splicing of genes commonly occurs in diverse diseases [35, 36], snoRNA levels are likely altered when host genes undergo abnormal alternative splicing, which is regulated by *cis*‐acting elements and *trans*‐acting factors. For example, the splicing factor FUS effectively regulates the expression of a subset of snoRNAs by influencing the splicing process [37]. Therefore, exploring the specific splicing factors that modulate the generation of key snoRNAs would be advantageous for elucidating the mechanisms underlying snoRNA‐associated diseases, as well as providing novel therapeutic targets. Furthermore, deep sequencing of small RNAs from supraspliceosomes, comprising four active native spliceosomes, revealed that approximately 25% of SNORDs expressed in HeLa cells were detected within supraspliceosomes [38]. These findings suggest that the generation of snoRNAs through splicing may differ from that of the canonical type, and further investigation of the underlying molecular mechanisms is required.

Besides to intronic snoRNAs, some snoRNAs are transcribed as independent genes, often by Pol II or III, these snoRNAs are called intergenic snoRNAs [26, 33]. For instance, the biogenesis of U3 snoRNA, which is transcribed by Pol II in both vertebrates and invertebrates, involves an endonucleolytic cleavage step during pre‐rRNA processing [39]. Another abundant snoRNA MRP has been reported to be transcribed by Pol III in both metazoa and plants [40]. Despite some progress made through bioinformatic analysis [41], the structure and function of independently transcribed snoRNA promoters remain largely unknown in humans.

Recently, several factors are also found contributing to the differential transcription of snoRNAs. Mutations in snoRNA genes can lead to changes in their expression levels, affecting their function and contributing to disease development (e.g., SNORA29 [42]; SNORNA U50 [43]). Changes in the copy number of genes containing snoRNAs can also impact their expression levels, resulting in differential transcription [44], for example, SNORA42 [45]. It is also important to note that while the majority of intronic snoRNAs are produced through the splicing process, certain intronic snoRNAs, such as U74, have been demonstrated to be generated via a splicing‐independent pathway that requires an extended external stem structure [46]. Therefore, the regulatory mechanisms underlying snoRNA biogenesis are indeed intricate, and a deeper understanding of these mechanisms will enhance our comprehension of snoRNA biology and contribute to elucidating the pathology of snoRNA‐related diseases.

## 3 Canonical Roles of snoRNAs

The snoRNAs are ncRNAs best known for directing chemical modifications on other RNAs, particularly rRNAs and snRNAs. Their canonical roles include guiding RNA modifications, such as 2′‐O‐methylation, pseudouridylation, and ac4C modification, as well as facilitating ribosome assembly [47].

### 3.1 RNA Modification

The most abundant modified nucleoside in human total RNA is pseudouridine (Ψ) [48, 49]. Notably, scaRNPs and snoRNAs play crucial roles in the modification of rRNA and snRNA, ensuring proper processing and maturation of RNA molecules within the cell through their interactions. snoRNAs not only guide the modification of rRNAs, tRNAs, and snRNAs, but also influence other RNAs in several ways. As mentioned above, C/D box snoRNAs (SNORDs) mainly facilitate 2′‐O‐methylation, while H/ACA box snoRNAs guide pseudouridylation [28, 29]. In C/D‐box snoRNAs, when the 5′ and 3′ ends fold into a stem structure, the C‐box (RUGAUGA, where R = A or G) and D‐box (CUGA) sequence motifs come into close proximity, forming a kink‐turn structure [50]. Most C/D box snoRNAs possess additional, less conserved C′ and D′ box motifs in the central region of the snoRNA. These snoRNAs guide modifications by forming base pairs with target RNAs through a short antisense element, typically 7–21 nt in length, located upstream of the D and/or D′ boxes. The structure of C/D box snoRNAs recruits associated proteins, such as Snu13, Nop56, Nop58, and the methyltransferase fibrillarin (FBL), which localize to the snoRNP complex [51, 52]. Binding to these proteins protects the snoRNA from degradation by exonucleases and plays a crucial role in its functions [53, 54]. Briefly, SNORDs guide 2′‐O‐methylation by base‐pairing with specific sequences in target RNAs and recruiting snoRNP complex containing the methyltransferase FBL. This interaction positions FBL precisely on the nucleotide to be modified, catalyzing the 2′‐O‐methylation step.

The antisense sequences of certain snoRNAs do not exhibit similarities to any known rRNA 2′‐O‐methylation sites, suggesting their potential involvement in directing 2′‐O‐methylation modifications to other RNAs such as mRNAs and tRNAs [55]. For example, C/D box RNPs can perform 2′‐O‐methylation on tRNA [56]. The 2′‐O‐methylation of the wobble cytidine at position C34 in the human elongator tRNA^Met^(CAT) is carried out through the combined action of a nucleolar protein and a CB‐specific box C/D RNP, which contain the SNORD97 and SCARNA97 box C/D 2′‐O‐methylation guide RNAs [57]. In a recent study by Lu et al. [58], it has been reported that C/D box SNORD104 promotes the 2′‐O‐methylation of PARP1 mRNA enhancing its stability. Additionally, the 2′‐O‐methylation of Pxdn mRNA, mediated by SNORDs, alters mRNA level and modulates protein translation [55]. Through this mechanism, SNORDs can regulate the stability, folding, and translation of both ribosomal and messenger RNAs, providing a crucial layer of posttranscriptional control. The antisense regions of H/ACA‐box snoRNAs consist of two short nucleotide sequences located within the internal loops of the 5′ and/or 3′ hairpin structures [59]. The 14th or 15th nucleotide upstream of the H/ACA box motifs pairs with the target RNA. Similar to C/D box snoRNAs, the H/ACA‐box‐associated proteins (dyskerin, Nhp2, Nop10, and Gar1) bind to H/ACA‐box snoRNA, forming snoRNPs that are essential for the of the rRNA pseudouridylation [60], Briefly, H/ACA box snoRNAs guide pseudouridylation by forming base pairs with the target RNA around the uridine to be modified and recruiting a specialized ensemble of proteins, including dyskerin (the pseudouridine synthase), NOP10, NHP2, and GAR1. This snoRNA–RNA interaction precisely positions dyskerin at the targeted uridine, facilitating the isomerization of uridine to pseudouridine [60, 61, 62].

It is believed that modifications introduced by snoRNPs provide protection to target RNAs from degradation by nucleases and expand the chemical space for RNA–RNA and RNA–protein interactions [63, 64]. Recent studies have revealed that snoRNAs are involved not only in 2′‐O‐methylation and pseudouridylation but also in the ac4C modification of RNA. Specifically, SNORD13 mediates single cytosine acetylation in 18S rRNA across human and zebrafish [65]. In addition to rRNAs, the ac4C modification is widely observed in other RNAs including tRNAs, mRNAs, and lncRNAs [66]. However, it remains unclear whether snoRNAs also contribute to regulating the ac4C modification in these RNAs and what the underlying regulatory mechanisms might be, warranting further investigation.

### 3.2 Assembly of Ribosomal Subunits

Ribosomes are large ribonucleoprotein complexes [67] composed of two distinct subunits. In prokaryotes, the 70S ribosome consists of a large 50S subunit and a small 30S subunit. The 50S subunit contains 23S rRNA, 5S rRNA, and approximately 34 proteins, serving as the catalytic center for peptide bond formation. The 30S subunit comprises 16S rRNA and about 21 proteins, primarily responsible for mRNA decoding and ensuring accurate tRNA–mRNA pairing during translation initiation. In contrast, eukaryotic 80S ribosomes are assembled from a 60S large subunit (LSU) and a 40S small subunit (SSU). The 60S subunit incorporates three rRNA species (28S, 5.8S, and 5S rRNAs) along with approximately 49 proteins. Beyond catalyzing peptide bond formation, this subunit plays additional roles in coordinating nascent polypeptide chain folding. The 40S subunit contains an 18S rRNA molecule and about 33 proteins, specializing in mRNA recognition through features such as 5′ cap structure identification and initiation codon selection [68, 69]. Ribosome biogenesis requires precise rRNA folding, ordered incorporation of ribosomal proteins, and tight regulation by assembly factors. In prokaryotes, rRNA gene clusters are transcribed into pre‐rRNA, which undergoes RNase III cleavage to generate discrete rRNA species. The 16S rRNA subsequently assembles with approximately 21 proteins in the cytoplasm to form the 30S SSU, while the 23S and 5S rRNAs combine with about 34 proteins to construct the 50S LSU. This assembly process, driven by GTPase‐mediated conformational adjustments, achieves structural maturation entirely within the cytoplasmic compartment. Eukaryotic ribosome assembly demonstrates greater complexity, involving multiple subcellular compartments. RNA Pol I in the nucleolus transcribes a large 45S pre‐rRNA precursor that is processed through endonucleolytic cleavage to yield 18S, 5.8S, and 28S rRNAs. The 40S SSU assembles in the nucleolus through coordinated interactions between 18S rRNA and approximately 33 proteins, while 60S LSU formation occurs in the nucleoplasm through sequential incorporation of 5.8S/28S/5S rRNAs with about 49 proteins. Both subunits undergo energy‐dependent structural remodeling through nucleocytoplasmic transport before achieving functional maturity in the cytoplasm, a process requiring over 200 assembly factors including chaperones, helicases, and ATP‐dependent RNA remodelers [70, 71].

snoRNAs perform multifaceted roles during ribosome biogenesis, including guiding pre‐rRNA folding, forming RNP substrates, facilitating RNA cleavage, directing base modifications, coordinating pre‐ribosomal subunit assembly, and mediating RNP particle export [1]. Among these, U3 snoRNA represents an essential nucleolar component critical for ribosome maturation, functioning as a structural chaperone to orchestrate proper folding of precursor 18S rRNA [72, 73]. Biochemical studies reveal a functional interdependence between U3 snoRNP complexes, Rrp5p helicase, and Rok1p ATPase—all three components being indispensable during early‐stage pre‐rRNA processing and assembly transitions [74]. Notably, the U3 snoRNP‐associated 50S particle has been identified as a SSU assembly intermediate that likely docks onto pre‐rRNA through cooperative interactions with RNA‐binding proteins (RBPs) nucleolin and RRP5, suggesting a molecular bridging mechanism during ribosome maturation [75]. During ribosomal subunit biogenesis, U3 snoRNA serves as the central architectural scaffold for 40S SSU assembly in eukaryotes. This ncRNA orchestrates the structural folding of precursor 18S rRNA within the nucleolus and recruits key proteins—including nucleolin, RRP5, and helicases such as Rok1p—to form the SSU processome, a dynamic assembly intermediate essential for guiding precise cleavage and maturation of the SSU [73, 75]. In contrast, 60S LSU maturation predominantly bypasses structural RNA chaperones like U3, instead relying on C/D box and H/ACA box snoRNAs to mediate canonical chemical modifications (2′‐O‐methylation and pseudouridylation) in 28S, 5.8S, and 5S rRNAs, thereby stabilizing LSU architecture and functional competence [70, 71]. This functional dichotomy reflects evolutionary divergence: prokaryotes, lacking snoRNAs entirely, depend solely on protein chaperones for ribosome assembly, whereas eukaryotes evolved snoRNAs to regulate the intricate nucleolar processing, quality control, and subunit trafficking required for their more complex ribosome biogenesis pathways.

In summary, the canonical functions of snoRNAs include not only 2′‐O‐methylation and pseudouridylation, but also the ac4C modification of RNA. Besides, snoRNAs play a crucial role in modulating ribosome assembly, thereby profoundly influencing gene translation. Interestingly, the snoRNAs are also involved in a variety of noncanonical functions and mechanisms, such as mRNA processing regulation, regulation of gene alternative splicing, the production of sdRNAs, and interaction with lncRNAs.

## 4 Noncanonical Functions of snoRNAs

### 4.1 mRNA Processing Regulation

Notably, snoRNAs play a role in regulating mRNA maturation by controlling the 5′ capping and 3′ poly(A) tail addition of mRNAs, which are crucial for preventing degradation by nucleases and maintaining mRNA stability [76]. It is well recognized that the 3′ end processing of mRNA is an essential step in gene expression. Studies have revealed that the majority of RNAs copurified with the 3′ mRNA processing complex are snoRNAs, which play a significant role in regulating mRNA 3′ processing [9]. Huang et al. [9] discovered that snoRNAs can directly bind to the Fip1 protein, a core component of this complex [9]. By doing so, snoRNAs inhibit the interaction between the Fip1 protein and the mRNA 3′ end poly(A) site, preventing excessive adenylation of the mRNA and thereby regulating the expression levels of different mRNAs. These snoRNAs can exert a decisive influence on transcription, translation efficiency, and cellular mRNA transport by affecting mRNA 3′ processing [77]. This discovery highlights the intricate regulatory network within the gene expression pathway, where ncRNAs like snoRNAs have emerged as key modulators. This suggests that mRNA plays a significant role in gene expression and regulation [78, 79, 80, 81, 82]. Recent research indicates that pseudouridylation, a modification mediated by snoRNAs, can indeed occur on mRNA targets [83]. This modification has the potential to regulate various aspects of mRNA function, such as stability, localization, and translation, thereby modulating the translation of the encoded proteins. Moreover, H/ACA RNPs are involved in the biogenesis of telomerase and can catalyze the pseudouridylation of RNA and spliceosomal RNA. snoRNAs, a type of H/ACA RNP, may indirectly influence the function or stability of telomerases via their role in RNA pseudouridylation [84]. The connection between telomerase and snoRNAs through the H/ACA RNPs further underscores the vital role of snoRNAs in cells. For instance, snoRNAs can guide specific modifications at targeted RNA sites. Song et al. [76] developed a method that uses snoRNAs as guiding tools to induce specific modifications at targeted RNA sites, thereby re‐editing disease‐associated aberrant mRNAs and enhancing gene expression. Most RNAs enriched in the mRNA 3′ end processing complex are snoRNAs. These findings indicate the diverse and intricate roles of snoRNAs in modulating mRNA processing.

Collectively, the canonical functions of snoRNAs include mediating RNA chemical modifications such as 2′‐O‐methylation, pseudouridylation, ac4C, and ribosome assembly. Beyond these established roles, snoRNAs exhibit noncanonical mechanisms to regulate gene expression and translation processes through regulating mRNA processing, gene alternative splicing, and epigenetic regulation including production of sdRNAs and interaction with lncRNAs. These canonical and noncanonical functions of snoRNAs highlight their crucial roles in extensively regulating gene expression, mRNA processing, and gene translation. However, aberrant regulation of these processes can lead to a variety of diseases, including cancer, neurological disorders, CVDs, immune‐inflammatory conditions, and aging, where snoRNAs actively contribute to disease pathogenesis and progression.

### 4.2 Regulation of Alternative Splicing

Alternative splicing, a critical mechanism in gene expression, enables a single gene to generate multiple protein isoforms through the selective inclusion or exclusion of exons from precursor messenger RNA(pre‐mRNA). This process is regulated by the spliceosome, a complex assembly of RBPs and snRNAs, which is further modulated by *trans*‐acting factors and *cis*‐acting elements [85]. Disruptions in alternative splicing have been implicated in a wide range of diseases, including cancer, neurological disorders, genetic conditions, immune dysfunctions, and CVDs [35, 86]. Aberrant splicing events can result in oncogenic protein isoforms, loss of tumor suppressor function, or the production of dysfunctional proteins, thereby disrupting essential cellular processes [87].

Besides to canonical *trans*‐acting factors and *cis*‐acting elements, snoRNAs also play a role in regulating alternative splicing [6, 88]. Some snoRNAs directly base‐pair with pre‐mRNA near splice sites, influencing exon inclusion or exclusion. For instance, HBII‐52 (SNORD115) regulates serotonin receptor 2C (HTR2C) splicing by promoting exon inclusion through base‐pairing to the alternatively spliced exon Vb in the Prader‐Willi syndrome (PWS) [89]. Furthermore, new splicing regulation targets for HBII‐52 have been identified, including DPM2, TAF1, RALGPS1, PBRM1, and CRHR1, based on sequence complementarity [90]. These findings suggest that HBII‐52 may serve as a potential therapeutic target for the treatment of PWS. Notably, intron‐hosted snoRNA, for example, snoRD86, acts as a cis‐acting snoRNA, regulating the splicing of host gene NOP56 pre‐mRNA [91]. A recent computational analysis of large‐scale human RNA–RNA interaction datasets by Bergeron et al. [92], suggests that numerous intronic snoRNAs may directly interact with their host transcripts, potentially modulating host gene splicing through base pairing with adjacent intronic sequences. Indirectly, snoRNAs may influence the modification of splicing factors, altering their activity. For instance, SNORA37 interacts with the cap methyltransferase 1 (CMTR1), enhancing its interaction with ELAVL1 and consequently promoting the nuclear retention in modulating alternative splicing of CD44, which drives gastric cancer progression [93]. However, the indirect influence of snoRNAs on the regulation of gene alternative splicing remains less well understood. The discovery of snoRNAs as regulators of alternative splicing introduces another layer of complexity to the already intricate process of gene expression and highlights the diverse functions of ncRNAs in both physiological and pathogenic bioprocesses.

### 4.3 snoRNA‐Derived Small RNAs

**FIGURE 1:**
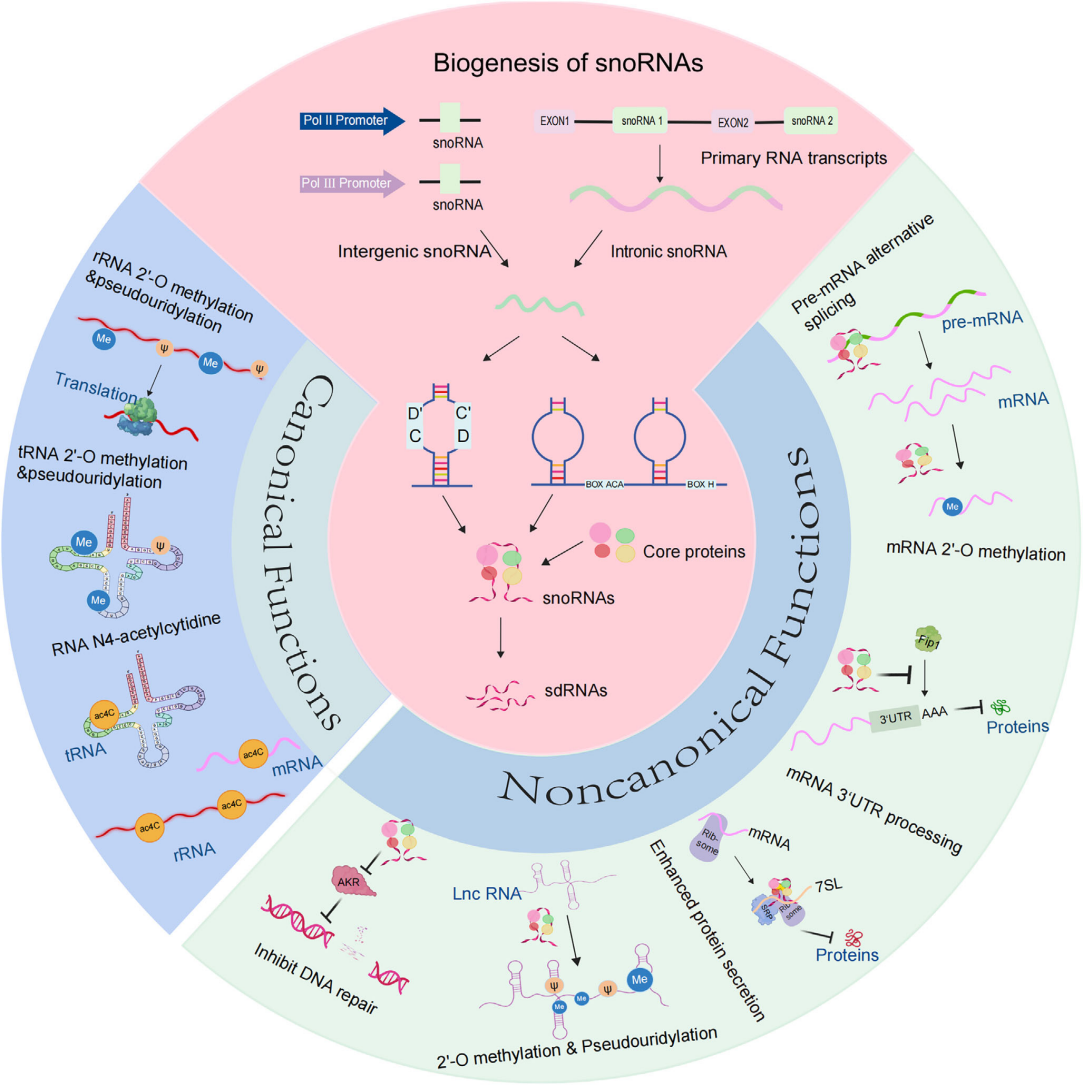
Overview of snoRNA biogenesis, classification, and canonical versus noncanonical functional roles. The biosynthesis of snoRNAs and diverse functions of snoRNAs. The majority of snoRNAs are derived from the intron region of genes and undergo maturation after the splicing process, the lariat RNA debranching and 5′/3′ trimming, besides to intronic snoRNAs, some snoRNAs are transcribed as independent genes, often by Pol II or III, these snoRNAs are called intergenic snoRNAs. snoRNAs exert their biological functions through not only 2′‐O methylation but pseudouridylation and N4‐acetylcytidine modifications on rRNAs, tRNAs and lncRNAs, influence the translation process, as well as RNA stability. Additionally, snoRNAs can affect mRNA 2′‐O methylation and 3′‐URT processing, regulating the translation efficiency and stability. snoRNAs are also capable of inhibiting DNA repair and enhancing protein secretion. snoRNAs, small nucleolar RNAs; sdRNAs, snoRNA‐derived small RNAs; lncRNAs, long noncoding RNAs; ac4C, N4‐acetylcytidine. Figure 1 was created with BioRender.

High‐throughput sequencing has revealed that snoRNAs are further processed to generate smaller fragments termed sdRNAs, and the expression patterns of these sdRNAs exhibit high evolutionary conservation across species [94]. sdRNAs can be classified into distinct categories based on their cellular origin and length characteristics. H/ACA box snoRNAs typically generate 20–24 nt fragments that are predominantly derived from their 3′ termini. In contrast, C/D box snoRNAs have been reported to produce two distinct fragment types: longer species exceeding 26 nt in length and shorter 17–19 nt fragments that primarily originate from the 5′ end [94, 95]. Although the functional significance remains unclear, emerging evidence has documented several novel functional possibilities associated with these processed fragments. Emerging research has demonstrated that sdRNAs, similar to miRNAs, can inhibit mRNA translation and stabilize pre‐mRNA [95, 96, 97, 98] (Figure 1). In addition to being well‐studied in cancer progression, as extensively reviewed [99, 100], sdRNAs have recently been shown to play crucial roles in chondrocyte senescence and osteoarthritis (OA) progression [101]. This suggests that sdRNAs derived from snoRNAs may also be critical for the progression of other diseases beyond cancer.

### 4.4 Interaction with lncRNAs

Unlike other ncRNAs, snoRNAs have a relatively well‐defined and specialized role in modifying other RNAs rather than directly regulating gene expression or interacting with mRNA, which is characteristic of miRNAs and lncRNAs [89, 102, 103, 104, 105, 106, 107, 108]. The early study was the first to propose that lncRNA GAS5 acts as the host gene for multiple snoRNAs [109]. The synergistic regulation between snoRNAs and their host ncRNAs (such as lncRNAs), exemplified by the collaborative role of host lncRNAs and snoRNAs in breast cancer metastasis, highlights the interaction between these molecules [25, 110]. Some introns can generate a distinct form of stable noncoding transcripts. snoRNAs are predominantly encoded within the introns of protein‐coding genes, from which they are processed and excised [111]. Certain intronic lncRNAs contain snoRNAs at both termini. These splicing‐derived RNA species, processed from introns and flanked by snoRNA structures at both ends, are specifically designated as sno‐lncRNAs. When an intron harbors two snoRNA genes, the cotranscriptionally assembled box C/D snoRNPs at both termini protect the intervening intronic sequences from exonucleolytic degradation following the debranching step during splicing, thereby facilitating sno‐lncRNA biogenesis. Notably, the C‐box element in the 5′‐terminal snoRNA and the D‐box motif in the 3′‐terminal snoRNA are critically required for the proper processing of sno‐lncRNAs. Given that the stabilization of sno‐lncRNAs is mediated by the formation of snoRNP complexes at both ends, rigorous comparative analyses have demonstrated that sno‐lncRNAs exhibit significantly extended half‐lives compared with expression‐matched mRNAs [111].

## 5 snoRNAs in Human Diseases

### 5.1 snoRNAs in Cancer

Numerous studies have demonstrated the close relationship between snoRNAs and various types of tumors. Among the primary tumors investigated, liver, lung, breast, prostate, colorectal, and meningiomas have garnered significant attention. These studies highlight the pivotal role of snoRNAs in tumor biology, emphasizing their potential as diagnostic, prognostic, and therapeutic targets for a diverse array of cancers. sdRNAs participate in the regulation of gene expression, thereby implicating snoRNAs in the development of various pathological conditions including cancer [99]. snoRNAs primarily engage in tumorigenesis by influencing phenotypes such as proliferation, migration, invasion, apoptosis, regulation of cell cycle functions, and alteration of the tumor microenvironment [112, 113, 114]. snoRNAs are believed to promote rRNA modifications, leading to dysregulation of housekeeping functions and subsequent cancer progression. Additionally, some snoRNAs promote cancer through noncanonical modifications, including, but not limited to, the methylation of 28S rRNA [53] and ubiquitination of GMPS [112].

**FIGURE 2:**
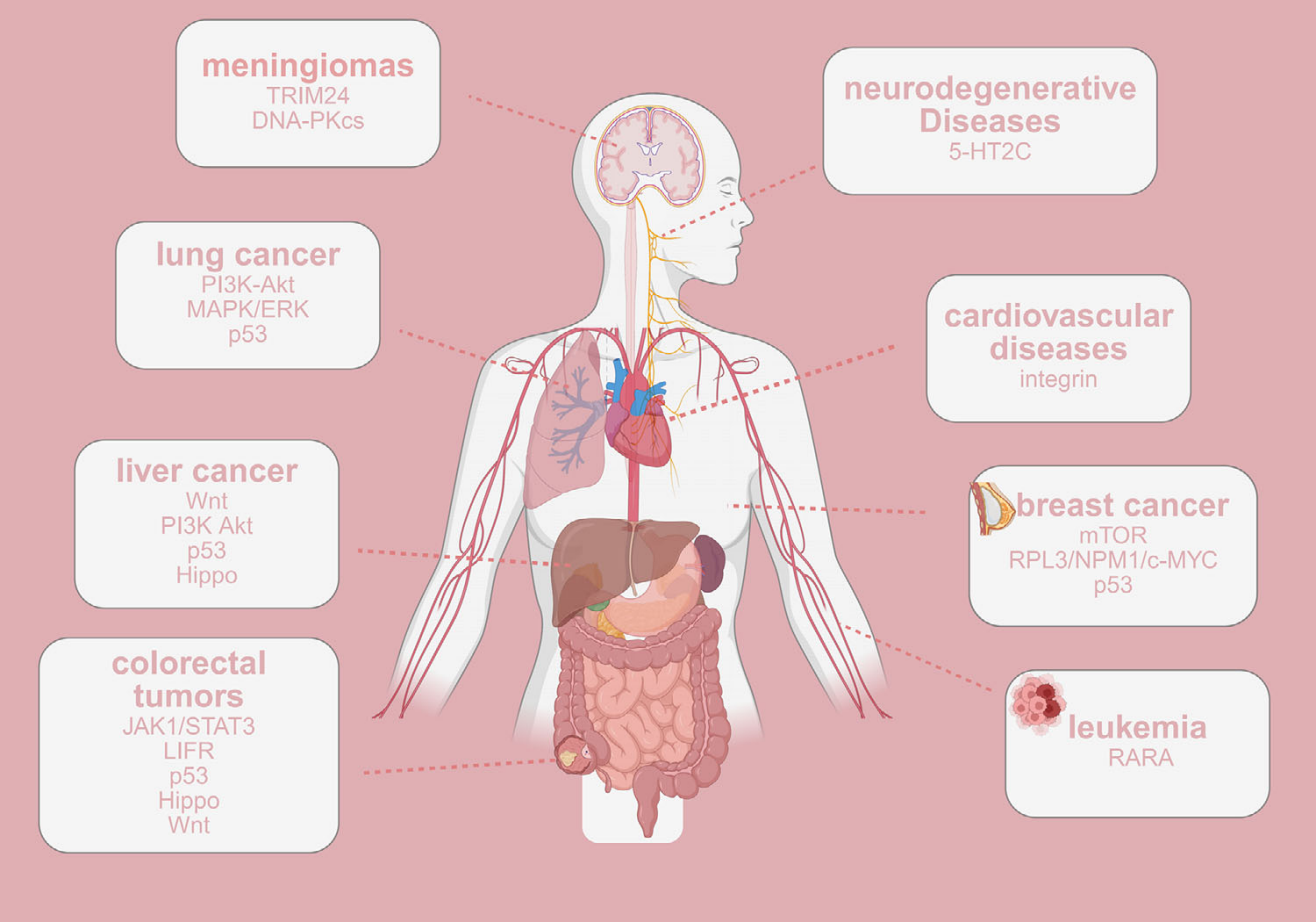
Dysregulated snoRNA‐related pathways in diseases. TRIM24, tripartite motif containing 24; DNA‐PKcs, DNA‐dependent protein kinase catalytic subunit; PI3K–Akt, phosphatidylinositol‐3‐kinase; MAPK, mitogen‐activated protein kinase; ERK, extracellular regulated protein kinases; JAK1/STAT3, the Janus kinase/signal transducer and activator of transcription; LIFR, leukemia inhibitory factor receptor; 5‐HT2C, 5‐hydroxytryptamine receptor 2C; mTOR, mammalian target of rapamycin; RPL3, ribosomal protein L3; NPM1, nucleophosmin; RARA, retinoic acid receptor alpha. Figure 2 was created with BioRender.

According to the Global Cancer Observatory, in 2022, liver cancer ranked sixth in terms of diagnosed cases among cancer‐related diseases and third globally in terms of cancer‐related mortality rates. In the same year, approximately 750,000 individuals died of liver cancer [115]. Studies have revealed the dual roles of specific snoRNAs in liver cancer. SNORD17 suppresses liver cancer cell growth through the p53 pathway [116], while SNORD53 promotes liver cancer by enhancing protein expression through CDK1 binding [117]. Additionally, the snoRNA ACA11 activates the PI3K–Akt pathway, facilitating cell growth, migration, and invasion in hepatocarcinogenesis [118]. Similarly, snoRNA U2‐19 participates in hepatocarcinogenesis by activating the Wnt signaling pathway [119] (Figure 2). These findings underscore the diverse and context‐dependent roles of snoRNAs in liver cancer and suggest their potential as therapeutic targets or biomarkers. A clinical study revealed that elevated SNORA23 expression was correlated with significantly reduced survival time, suggesting a close association between SNORA23 and liver cancer prognosis [120]. Furthermore, researchers have identified SNORA11, SNORD124, SNORD46, and SNORD52 as pivotal factors in the diagnosis and prognosis of liver cancer [117, 121]. Recent studies have shown that SNORD88B is highly expressed in liver cancer stem cells (CSCs) and liver cancer samples, where it promotes the binding of XRCC5 to the STK4 promoter, thereby inhibiting STK4 transcription. STK4 is a key kinase involved in the Hippo signaling pathway, and its inhibition leads to the inactivation of this pathway (Figure 2). Consequently, YAP1 translocates to the nucleus and acts as a transcriptional regulator promoting cell proliferation and self‐renewal. SNORD88B drives self‐renewal of liver CSCs and accelerates hepatocellular carcinoma (HCC) progression through a nonclassical mechanism. Furthermore, the combined use of SNORD88B antisense oligonucleotides (ASOs) and the MST1 agonist adapalene was found to have a synergistic antitumor effect, demonstrating enhanced anticancer activity and improved survival rates in mice [122]. These findings provide a significant scientific basis for the development of new therapies targeting liver CSCs. Additionally, research suggests that the abnormal expression of SNORD12B, SNORA63, and SNORD14E could serve as novel noninvasive biomarkers for HBV‐associated HCC [123]. These findings underscore the clinical relevance of snoRNAs in the management and prognostic assessment of liver cancer.

Despite a gradual decline in the number of diagnosed cases, lung cancer still maintains its top position in terms of incidence and mortality rates among related diseases [115, 124]. SNORA80E has been identified as a promoter of cell proliferation and inhibitor of apoptosis in lung cancer through the p53 pathway, thereby facilitating lung cancer growth [125]. Additionally, studies have demonstrated that SNORA71A regulates the cell cycle and epithelial–mesenchymal transition via the MAPK/ERK pathway in lung cancer [126] (Figure 2). These findings highlight the significant roles of SNORA80E and SNORD71A in the pathogenesis and progression of lung cancer, providing potential targets for therapeutic interventions. Furthermore, SNORA38B can impact the occurrence of non‐small cell lung cancer (NSCLC) by reducing the infiltration of CD3^+^ CD8^+^ T cells and increasing the activity of CD4^+^ FOXP3^+^ T‐reg cells in the tumor microenvironment [114]. Studies have revealed that knockdown of SNORA47 markedly suppresses the development of NSCLC by inhibiting the PI3K–Akt signaling pathway [127] (Figure 2). The potential clinical utility of SNORD33 and SNORD76, which are highly expressed in the plasma of patients with NSCLC, as auxiliary diagnostic tools warrants further investigation [128]. These findings highlight the therapeutic potential of targeting snoRNAs, such as SNORA47, and the possibility of SNORD33 and SNORD76 serving as biomarkers for NSCLC diagnosis; however, additional research is needed to validate their clinical applicability.

Breast cancer (BC) is the most commonly diagnosed cancer and the second leading cause of cancer‐related death in women [129]. Consequently, the identification of potential snoRNA biomarkers is crucial for diagnosis and treatment targeting. Dysregulation of U50 and overexpression of snoRNAs in FBL and C/D box have been observed in breast and prostate cancers [43, 130, 131]. FBL, an enzymatic component of C/D box snoRNPs, has been implicated in tumorigenesis by inhibiting p53 function (Figure 2). Furthermore, studies have highlighted the role of H/ACA box small nucleolar RNA7b in influencing the migration and invasion of BC cells, suggesting that specific snoRNAs are potential prognostic markers of BC [110, 132]. These findings underscore the significance of snoRNAs in the pathogenesis and prognosis of breast and prostate cancers and highlight their potential as therapeutic targets or prognostic indicators. SNORD50A/B binding to GMPS leads to the ubiquitination of p53, thereby compromising its ability to induce apoptosis [112]. Recent studies have shown that SNORA51 enhances the characteristics of breast CSC characteristics by regulating the RPL3/NPM1/c‐MYC pathway (Figure 2). Notably, high SNORA51 expression is associated with poor prognosis, overall survival, and disease‐free survival. SNORA51 expression is also closely related to lymph node status, estrogen receptor status, Ki‐67 index, histological grade, and TNM stage [133]. Therefore, SNORA51 may serve as an important biomarker and potential therapeutic target, offering new insights into the diagnosis and treatment of BC and potentially providing valuable implications for research on other malignant tumors. Other studies have shown that snoRNAs (SNORD16, SNORA73B, SCARNA4, and SNORD49B) are stably expressed and significantly upregulated in BC tissues and patient plasma. Their strong diagnostic potential suggests that they can be used as noninvasive biomarkers for BC detection [134]. Furthermore, research has found that snoRNAs could serve as potential biomarkers for assessing local and regional metastasis in patients with BC, and for predicting prognosis [135]. More importantly, studies have shown that U50A regulates BC development and drug resistance by influencing the expression of mTOR, thereby modulating the mTOR signaling pathway [136]. U50A could be an important indicator for assessing the response of patients with BC to mTOR inhibitors, such as everolimus (Figure 2). The mechanism of action of U50A provides a theoretical foundation for the development of novel therapeutic strategies.

snoRNAs are also closely related to colorectal tumors. U50 expression has been found to be downregulated in colorectal tumors [137], while SNORD1C has been implicated in tumorigenesis through activation of the Wnt signaling pathway [138]. Moreover, SNORA21 participates in cell proliferation and enhances tumor invasiveness by regulating the Hippo and Wnt signaling pathways. SNORA24, on the other hand, can affect P53 function, promoting cell cycle progression and proliferation. This underscores its potential value as a biomarker and therapeutic target for patients with CRC [113, 139, 140]. These findings indicate that the abnormal expression of snoRNAs is closely related to the occurrence and progression of colorectal cancer. Recent studies have shown that SNORA28 plays a significant role in CRC growth and radioresistance by regulating LIFR and STAT3 signaling pathways. SNORA28 recruits bromodomain‐containing protein 4, which increases the acetylation level of H3K9 in the promoter region of LIFR, thereby stimulating LIFR transcription. This activation subsequently triggers the JAK1/STAT3 signaling pathway, ultimately enhancing CRC cell proliferation and radioresistance (Figure 2). This provides a new perspective on the role of snoRNAs in CRC pathogenesis and suggests that snoRNAs could be potential therapeutic targets for improving the efficacy of CRC radiotherapy [141, 142].

In meningiomas, the expression of h5sn2 snoRNA is significantly lower than that in the normal brain tissue [143]. U3 snoRNAs, through their interactions with PHAX, TRIM24, and DNA‐PKcs, not only promote ribosome biogenesis, but also drive malignant transformation, similar to epithelial‐type glioblastoma, by regulating epigenetic modifications and transcription factor networks [144] (Figure 2). Studies on gastric tumors have suggested potential roles for snoRNAs such as SNORA42, SNORA74A, and SNORD10 in gastric tumor pathogenesis [145]. The specific mechanisms through which these snoRNAs contribute to tumorigenesis require further investigation to ascertain their potential as therapeutic targets.

snoRNAs also play a crucial role in diseases of the blood system. The development of leukemia requires enhanced self‐renewal, a process induced by oncogenic genes; however, the underlying molecular mechanisms remain poorly understood. Studies suggest that key factors driving leukemia stem cell activity include C/D box snoRNAs and rRNA 2′‐O‐methylation. The overall depletion of C/D box snoRNAs and loss of rRNA 2′‐O‐methylation diminish the self‐renewal potential of leukemia cells. Specifically, genomic deletion of C/D box snoRNAs, such as SNORD14D or SNORD35A, inhibits the clonal formation of leukemia cells in vitro and delays leukemia onset in vivo, indicating that the induction of C/D box snoRNA/RNP function constitutes an important pathway in leukemogenesis [146].

In 2012, Teittinen et al. [147] analyzed the expression of snoRNAs in different leukemia cell lines using sequencing technology and validated their expression profiles [147]. Their research focused on investigating the differential expression of snoRNAs in various leukemia cell subsets including acute myeloid leukemia, pre‐B acute lymphoblastic leukemia (pre‐B‐ALL), and T cell acute lymphoblastic leukemia (T‐ALL). Their findings revealed significant differences in snoRNA expression between the T‐ALL and pre‐B‐ALL cell subsets. Specifically, they identified 46 snoRNAs that were differentially expressed, and further validation confirmed the differential expression of four specific snoRNAs: SNORD49A, SNORD55, SNORD105, and SNORD110 [147, 148]. These results provide valuable insights into the potential roles of snoRNAs in leukemia pathogenesis, particularly in different leukemia subtypes. Understanding the specific snoRNAs associated with each subtype may aid in the development of targeted diagnostic and therapeutic strategies for patients with leukemia. Further research in this area may reveal additional snoRNAs and shed light on their functional significance in leukemia. These findings highlight the intricate relationship between snoRNAs and leukemia, particularly acute promyelocytic leukemia (APL), and shed light on their potential role in disease pathogenesis and treatment responses. In patients with APL treated with all‐trans retinoic acid (ATRA), it was found that ATRA directly targets the PML–RARA fusion gene, leading to cellular differentiation. Concomitantly, there is a reduced expression of the snoRNA gene cluster SNORD112‐114 located in the dlk1–dio3 locus during this differentiation process [149]. This suggests a potential regulatory role for snoRNAs in APL differentiation and warrants further investigation into their precise mechanisms of action. Moreover, studies have implicated the SNORD112‐114 gene cluster at the dlk1–dio3 locus in leukemia and stem cell pluripotency [150]. This suggests a broader involvement of snoRNAs in the pathogenesis of leukemia and highlights their potential as therapeutic targets or biomarkers for leukemia management.

In 2017, Zhou et al. [146] demonstrated the importance of box C/D snoRNA/RNP formation and rRNA 2′‐O‐methylation in the clonogenic ability of leukemia cells in vitro and their survival in vivo [146]. This underscores the significance of snoRNAs in leukemia cell biology and the potential relevance of targeting snoRNA‐related pathways in therapeutic interventions. Overall, these findings collectively underscore the importance of snoRNAs in leukemia biology and treatment responses, suggesting avenues for further research and potential therapeutic strategies targeting snoRNA‐related pathways in leukemia management. Insights provided by recent studies underscore the critical role of snoRNAs in the pathogenesis of leukemia, particularly in leukemia stem cell self‐renewal and hematopoiesis [151]. The deletion of snoRNAs, such as SNORD34, SNORD35A, SNORD43, and SNORD104, leads to disturbances in rRNA methylation and a decrease in the repair speed of damaged proteins in cells. This highlights the importance of snoRNAs in the maintenance of cellular homeostasis and integrity. Furthermore, snoRNAs have emerged as common downstream targets of oncogene regulation associated with the self‐renewal of leukemia stem cells [146]. Their dysregulation contributes to aberrant proliferation and survival of leukemia cells. Recent studies have elucidated the specific expression patterns of snoRNAs involved in the development and lineage commitment of human hematopoiesis. For example, the expression of SNORD42 is significantly enhanced in leukemic cells compared with that in CD34 progenitor cells, monocytes, and granulocytes. SNORD42A knockdown inhibits cell proliferation, and its deletion is associated with reduced ribosomal protein translation and decreased leukemia cell volume [152]. The loss of SNORD118 leads to a decrease in the number of cells in the G0/G1 phase and an increase in the number of cells in the S phase, indicating that its antiproliferative effect is mediated through cell cycle arrest. Its loss is also associated with widespread changes in chromatin accessibility, primarily occurring in intergenic regions and introns and affecting genes such as RARA and CDK14 (Figure 2). These data suggest that SNORD118 and SNORD3A play important roles in the maintenance of leukemia cell proliferation. Given its minimal effect on healthy hematopoiesis, SNORD118 may be a potential therapeutic target [148]. In summary, snoRNAs exert a multifaceted influence on leukemia pathogenesis, affecting various cellular processes, including rRNA modification, protein repair, and cell proliferation. Understanding the roles of snoRNAs in leukemia biology holds promise for the development of novel therapeutic strategies targeting these molecules for leukemia treatment.

Myelodysplastic syndromes (MDSs) are heterogeneous myeloid clonal disorders that originate from hematopoietic stem cells [153]. One important factor implicated in MDS pathogenesis is the 1q21.1 deletion (1ddx41 mutation), which has been associated with MDS development and progression [154]. The interplay between snoRNAs and MDS, particularly 1q21.1, warrants further investigation. Understanding how snoRNAs, including SNORD115 and SNORD116, influence gene expression and cellular processes relevant to MDS pathogenesis could provide valuable insights into disease mechanisms and potential therapeutic targets. Various studies highlight the critical role of DDX41 protein and its mutations in the pathogenesis of MDS, shedding light on the underlying mechanisms involving snoRNAs. Frameshift mutations at specific sites, such as D52 and D140, in the DDX41 protein have been implicated in the inactivation of DDX41, contributing to the development of MDS [154, 155, 156, 157, 158]. These mutations disrupt the normal function of DDX41, which is involved in various cellular processes including RNA metabolism and surveillance. Furthermore, mutations or deletions in DDX41 lead to abnormal snoRNA expression, resulting in abnormalities in ribosome assembly and protein synthesis [155]. This highlights the regulatory role of snoRNAs in the pathogenesis of MDS, indicating their involvement in the dysregulation of cellular processes essential for hematopoiesis. Understanding the interplay among DDX41 mutations, snoRNA dysregulation, and MDS development is crucial for elucidating the molecular mechanisms underlying this disorder. It also offers potential avenues for therapeutic interventions targeting snoRNA‐related pathways to mitigate the effects of DDX41 mutations and improve outcomes in patients with MDS.

### 5.2 snoRNAs in Neurodegenerative Diseases

Neurodegenerative diseases (NDDs) are disorders that affect the central nervous system (CNS) and are characterized by progressive loss of neuronal tissue [159]. NDDs present a significant challenge owing to their progressive nature and irreversible damage to neuronal tissue. Early diagnosis is crucial for effective treatment, as delayed diagnosis can limit the efficacy of available therapies [160]. Magnetic resonance imaging (MRI) is one of the most utilized neuroimaging techniques for diagnosing and monitoring various NDDs, including Alzheimer's disease (AD) and dementia with Lewy bodies. It provides detailed images of the brain's structure and can reveal characteristic patterns associated with these diseases. Studies have underscored MRI's importance in aiding accurate diagnosis and tracking disease progression over time. By leveraging MRI technology, healthcare professionals can potentially detect NDDS at earlier stages, allowing timely intervention and improved patient outcomes [161, 162, 163]. MiRNAs have emerged as prominent single‐stranded RNA biomarkers that have been investigated in diverse disease contexts, including cancer, aging, and NDDs. Various studies elaborate on the roles of miRNAs in these pathological processes, emphasizing their potential as diagnostic or prognostic markers, as well as therapeutic targets [164, 165, 166]. In contrast, snoRNAs predominantly target rRNAs to facilitate modifications crucial for ribosomal function [167]. snoRNAs can influence the alternative splicing of pre‐mRNAs, thereby contributing to the regulation of gene expression. Studies have investigated these specific functions and their implications in cellular processes [103]. Both miRNAs and snoRNAs represent fascinating areas of molecular biology research and hold significant promise for elucidating disease mechanisms, identifying biomarkers, and developing novel therapeutic strategies. Mutations in the SNORD118 allele, encoding small nucleolar RNA U8 within snoRNAs, affect the 3′ end processing of precursor U8, leading to the development of leukoencephalopathy with calcifications and cysts (LCC) [168].Labrune syndrome, also known as leukoencephalopathy with brainstem and spinal cord involvement and lactate elevation, is a rare neurological disorder characterized by abnormalities in the white matter of the brain, typically accompanied by calcifications and cysts. Labrune et al. [169] reported three children with Labrun syndrome, noting cognitive decline and the presence of spastic seizures alongside extrapyramidal and cerebellar symptoms across all three cases. The differential regulation of specific C/D box snoRNAs, such as E307 and E470, before the onset of AD in mouse models suggests their potential as early diagnostic markers of the disease. However, this differential expression ceases after the formation of beta‐amyloid plaques, indicating the importance of snoRNA expression alterations in the early stages of Alzheimer's pathology [170]. Furthermore, maternal alcohol consumption during pregnancy has been associated with changes in snoRNA levels, including alterations in DNA methylation, miRNA levels, and snoRNA levels, such as an increase in SNORD115 and decrease in SNORD116 in brain cells during fetal development [171]. These changes may contribute to the pathogenesis of fetal dysplasia associated with maternal alcohol consumption. Understanding the molecular mechanisms underlying these disorders and the involvement of RNA molecules such as snoRNAs provides valuable insights into their pathogenesis and potential avenues for diagnosis and treatment. These studies highlight the potential of snoRNAs as biomarkers for the early diagnosis of neurological diseases, specifically LCC. Jenskinson et al. [172] sequenced biological samples from 40 patients with LCC over 12 years and identified biallelic mutations in the SNORD118 fragment on chromosome 17 as a potential cause of LCC. Crow et al. [168] expanded this research by observing and analyzing data from 64 patients with LCC, revealing 44 different types of SNORD118 mutations that may contribute to the disease. These findings highlight the importance of snoRNAs in the pathogenesis of neurological disorders and their potential utility as diagnostic biomarkers. Early diagnosis facilitated by the identification of snoRNA mutations can enable timely intervention or treatment, potentially limiting the progression of these diseases. By leveraging snoRNAs as diagnostic markers, clinicians may improve their ability to detect neurological diseases at early stages, when interventions are most effective. Understanding the genetic basis of these disorders can pave the way for targeted therapies aimed at addressing specific molecular abnormalities associated with snoRNA mutations.

The 5‐HT2C receptor has been implicated in the pathogenesis of major depressive disorder (MDD) and schizophrenia (SCZ), as evidenced in multiple studies [173, 174, 175, 176, 177]. The regulation of 5‐HT2C mRNA expression by specific snoRNAs, such as hbi‐52 and hbi‐36, has been identified [178]. This suggests a potential mechanism through which snoRNAs influence mental health disorders by modulating serotonin receptor expression (Figure 2). Furthermore, studies in monozygotic twins with SCZ have revealed hypermethylation of specific regions of the snoRNAs SNORD115 and SNORD116 [179]. Additionally, SNORA69 alters the structure of rRNA by increasing the pseudouridylation levels of 5.8S rRNA or 18S rRNA. This modification affects the binding ability of rRNA with other molecules, such as tRNA and mRNA, thereby impacting the translation process and proteomic profile within the cell. This suggests that SNORA69 may be a potential biomarker for predicting MDD; however, further empirical research is needed to support this hypothesis [180]. Collectively, these findings highlight the involvement of snoRNAs in epigenetic regulation associated with SCZ and other mental health‐related disorders. Moreover, these findings underscore the intricate interplay between snoRNAs and the serotonin system in the pathogenesis of mental health disorders, such as MDD and SCZ. Understanding these molecular mechanisms may offer insights into novel therapeutic strategies and diagnostic biomarkers for these complex conditions.

Research conducted by Bieth et al. [181] and Tan et al. [182] has shed light on the crucial role of the SNORD116 gene cluster in the pathogenesis of PWS, a complex genetic disorder characterized by neurodevelopmental abnormalities [182, 183]. Adhikari et al. expanded research into PWS, highlighting the cognitive impairments associated with SNORD116 mutations and investigating learning and memory behaviors in a mouse model. Their findings suggest that the SNORD116± mutant mouse model exhibits differences in learning and memory behaviors compared with SNORD116+/+ control mice. Specifically, SNORD116+/+ mice display defects in novel object recognition, location memory, and tone cue fear conditioning, indicating impaired learning and memory [184]. This study highlights the importance of animal models for understanding the clinical manifestations of PWS, particularly cognitive impairment. The SNORD116± mutant mouse model, characterized by learning and memory deficits, offers a valuable tool for studying the neurological aspects of PWS and potentially for developing therapeutic interventions to address cognitive impairments associated with the syndrome. Bieth et al. [181] reported the case of a female PWS patient with a paternal deletion in the SNORD116 gene cluster, providing evidence for the involvement of SNORD116 in the development of PWS. This deletion disrupted the normal function of SNORD116 and contributed to the manifestation of PWS symptoms. Subsequently, Tan et al. [182] presented the rare case of a 17‐year‐old patient with PWS, further emphasizing the significance of paternal copy loss of SNORD116 in determining the clinical features of PWS. This suggests that the absence or dysfunction of SNORD116, inherited paternally, is a key determinant in the development of PWS phenotypes. These findings highlight the critical role of the SNORD116 gene cluster, particularly its paternal copy, in PWS pathogenesis. Understanding the molecular mechanisms underlying SNORD116 dysfunction may provide insights into the pathophysiology of PWS and potentially inform the development of targeted therapies for this complex disorder. The finding indicate a relationship between snoRNAs, particularly SNORD115 and SNORD116, and downstream gene expression in HEK 293T cells. SNORD116 upregulation alters the expression of over 200 genes, primarily mRNA transcripts, suggesting a potential regulatory role of SNORD116 in gene expression [185]. Additionally, the expression of SNORD116 is influenced by the upregulation of SNORD115, indicating potential interactions or regulatory mechanisms between these two snoRNAs. Furthermore, research has found that during anesthesia, propofol can affect the nervous system through interactions between RARα, SNHG1, and Bdnf [186]. Whether this effect is mediated by snoRNA fragments of SNHG1 requires further investigation.

### 5.3 snoRNAs in CVDs

**FIGURE 3:**
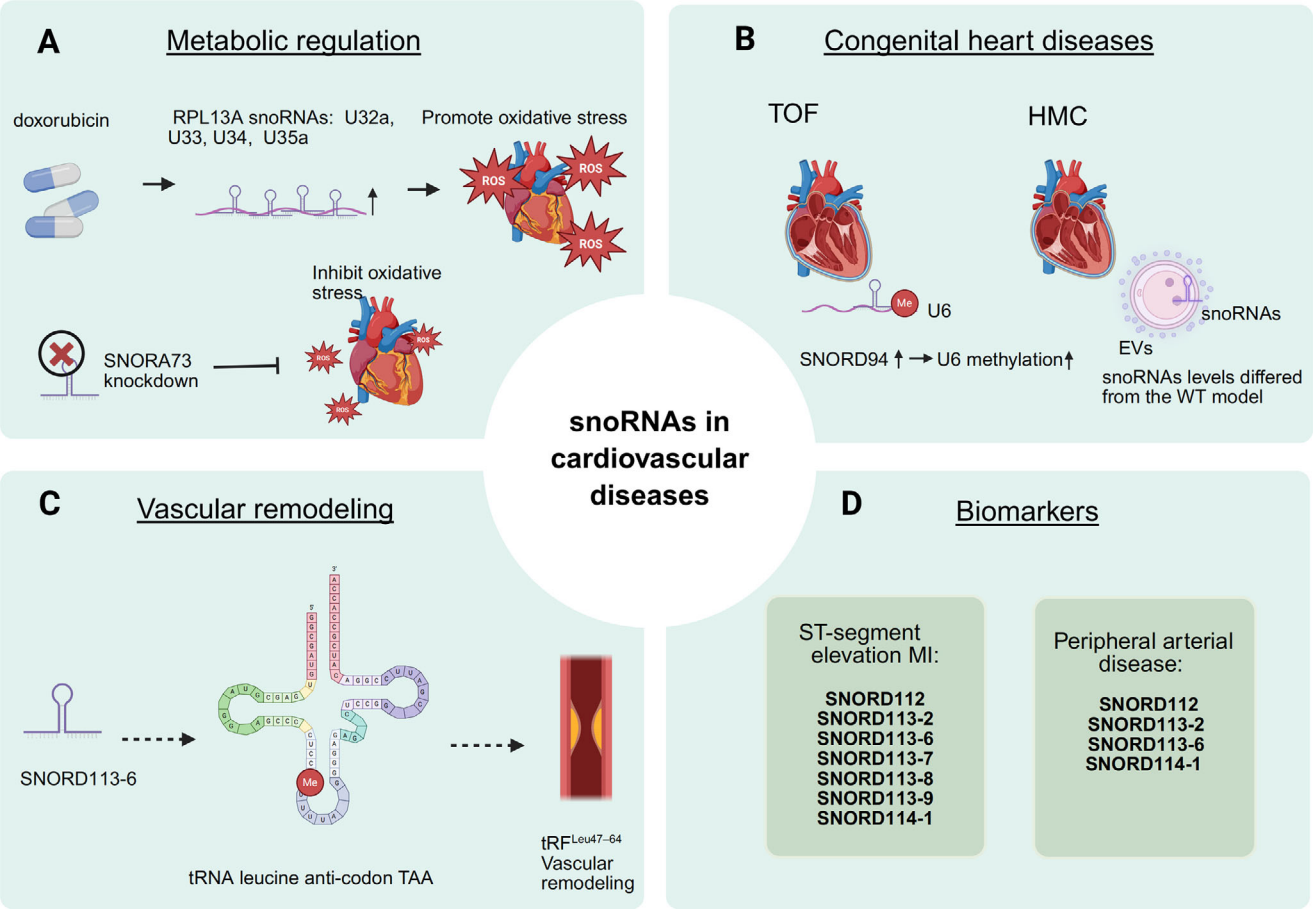
SnoRNAs play a crucial role in the development and progression of cardiovascular diseases. (A) Intronic snoRNAs, including U32a, U33, U34, and U35a, which are derived from the rpl13a gene, mediate doxorubicin‐induced oxidative stress and contribute to the elevation of ROS levels in heart tissues; knockdown of SNORA73 could prevent oxidative stress. (B) In the myocardium of infants with heart defects, SNORD94 levels directly influence the methylation levels at its target region in U6; Compared to wild‐type controls, EVs isolated from HCM models exhibited altered snoRNA levels. (C) SNORD113‐6‐mediated 2′‐O‐methylation of tRNA leucine anticodon TAA [tRNALeu(TAA)] prevented its fragmentation and the formation of tRFLeu47–64 in vascular remodeling. (D) SNORD112, SNORD113‐2, SNORD113‐6, SNORD113‐7, SNORD113‐8, SNORD113‐9, and SNORD114‐1 were significantly upregulated in ST‐segment elevation MI; SNORD112, SNORD113‐2, SNORD113‐6, and SNORD114‐1 were significantly upregulated in peripheral arterial disease. U32a, snoRD32A; U33, snoRD33; U34, snoRD34; U35a, snoRD35A; ROS, reactive oxygen species; TOF, tetralogy of Fallot; HMC, hypertrophic cardiomyopathy; WT, wild type; EV, extracellular vesicles; MI, myocardial infarction. Figure 3 was created with BioRender.

CVD are a leading cause of mortality worldwide. Extensive research has shown differential expression of snoRNAs in CVD, indicating that snoRNAs may play a role in their occurrence and progression. In 2019, CVD accounted for approximately one‐third of global deaths, with 9.69 million deaths in males and 8.9 million deaths in females [187]. CVD encompasses a range of conditions affecting the heart and blood vessels, with risk factors including genetic predisposition, lifestyle choices (e.g., diet, smoking, and physical activity), and obesity. Research indicates that snoRNAs can be reliably detected in the plasma and serum, and alterations in blood snoRNA levels have been associated with different stages of disease development in cardiovascular conditions. This suggests their potential utility as biomarkers for diagnosing and monitoring CVD [128, 188]. Using snoRNAs as biomarkers, clinicians can improve their ability to assess CVD risk, diagnose conditions earlier, and monitor disease progression more effectively. Additionally, understanding the roles of snoRNAs in cardiovascular pathophysiology could lead to the development of novel therapeutic strategies targeting specific molecular pathways implicated in CVD. The expanding body of literature linking snoRNAs to various CVD, including congenital heart disease (CHD), coronary heart disease, myocardial infarction (MI), and heart failure, provides valuable insights into the role of snoRNAs in cardiovascular pathophysiology [189, 190, 191]. These findings not only deepen our understanding of the molecular mechanisms underlying these diseases but also offer potential avenues for the development of novel disease biomarkers. Moreover, snoRNAs have been implicated in cardiometabolic diseases (CMDS), including doxorubicin cardiotoxicity [192, 193, 194]. Studies have elucidated the functions of snoRNAs in the regulation of metabolic stress responses in mammalian cells. For instance, RPL13A snoRNAs has been shown to accumulate in the cytoplasm during oxidative stress induced by doxorubicin (Figure 3). This accumulation is dynamically regulated by NADPH oxidase, suggesting a coordinated response of snoRNAs to environmental stressors [195]. Furthermore, Sletten et al. [196] demonstrated that the knockdown of snoRNA SNORA73 in vivo could prevent oxidative stress, highlighting the potential therapeutic implications of targeting snoRNAs in mitigating oxidative stress‐related damage. These studies collectively underscore the diverse roles of snoRNAs in cardiovascular and metabolic diseases, ranging from serving as potential biomarkers for disease diagnosis and prognosis to actively participating in cellular responses to stress. Further research in this area holds promise for advancing our understanding of disease mechanisms and for identifying novel therapeutic targets.

The role of snoRNAs in CMDS, including CHDs such as tetralogy of Fallot (TOF) and CHD, is becoming increasingly recognized in medical research (Figure 3). In TOF, studies comparing the expression of snoRNAs in the right ventricular myocardium of affected infants with that of normal fetal and infant hearts revealed significant differences in the expression of 135 snoRNAs, including 12 scaRNAs [189]. Additionally, Ogren et al. [197] reported elevated methylation of the SNORD94 target region on U6 in the right ventricular myocardial tissue of TOF infants compared with that in control groups. These findings underscore the importance of snoRNAs in the pathogenesis of CHDs such as TOF. Early diagnosis of CHD remains challenging; however, studies, such as the Framingham Heart Study, have shed light on the potential of extracellular RNAs (exRNAs), including snoRNAs, as biomarkers of the disease [198]. The Framingham Heart Study identified over 1,000 human exRNAs, including miRNAs, snoRNAs, and piRNAs, highlighting the diverse repertoire of circulating RNA molecules and their potential relevance in cardiovascular health [190]. Collectively, these findings suggest that snoRNAs may serve as valuable therapeutic targets and biomarkers for CMDS, offering new insights into disease mechanisms and potential avenues for early diagnosis and intervention.

The relationship between snoRNAs and coronary heart disease, particularly ST‐segment elevation MI (STEMI), is an area of active research. A study reported by Schena et al. [199] identified seven snoRNAs (SNORD112, SNORD113‐2, SNORD113‐6, SNORD113‐7, SNORD113‐8, SNORD113‐9, and SNORD114‐1) in the plasma of patients with STEMI after admission (Figure 3). Interestingly, SNORD113‐2 was found to be upregulated twofold in the plasma from day 4 to day 30 after admission in patients with STEMI, suggesting its potential as a biomarker for STEMI progression. Although the expression levels of the remaining snoRNAs changed over time, the changes were not statistically significant. Furthermore, Schena et al. [199] observed that treatment with transforming growth factor beta to activate fibrotic signaling and then treated with human cortical bone stem cell‐derived exosomes (hCBSC‐Dexos) reduced snoRNA levels in normal human ventricular cardiac fibroblasts. These findings suggest that snoRNAs present in CBSC‐dEXOs may influence protein translation and reduce fibroblast activity, highlighting the potential mechanism through which exosomal snoRNAs may affect cardiac function. These studies underscore the potential diagnostic and therapeutic implications of snoRNAs in coronary heart disease, particularly in the context of acute events such as STEMI. Further exploration of the roles of snoRNAs in cardiovascular pathophysiology could lead to the development of novel biomarkers and therapeutic strategies to improving patient outcomes in coronary heart disease. The identification of a large gene cluster on human chromosome 14 (14q32) encoding three lncRNAs, 41 snoRNAs, and 54 miRNAs suggests that a complex regulatory network is involved in CVD. Single‐nucleotide polymorphisms in the 14q32 snoRNA cluster are associated with heart failure, indicating the potential significance of these snoRNAs in cardiovascular pathophysiology [200]. Moreover, snoRNAs in 14q32 independently regulate CVD, emphasizing their unique role in cardiac health [191]. For instance, van Ingen et al. [201] highlighted the specific roles of snoRNAs such as SNORD113‐6/af357425 in the integrin signaling pathway and arterial fibroblast function. These snoRNAs are involved in processing/splicing pre‐mRNA and 2′‐O methylation of mRNA, ensuring the stability of mRNA expression [201]. These findings suggest that snoRNAs in 14q32 play crucial roles in the pathogenesis and regulation of CVD (Figure 2).

Future cardiovascular research should focus on these snoRNAs to better understand their specific functions and their potential as therapeutic targets for the management of cardiovascular disorders. The specific role of tRF^Leu47–64^ in vascular remodeling and CVD remains unclear. However, only some aspects of the function of SNORD113‐6/af357425 have been elucidated, SNORD113‐6‐mediated 2′‐O‐methylation of tRNA leucine anticodon TAA [tRNALeu(TAA)] prevented its fragmentation and the formation of tRF^Leu47–64^ in vascular remodeling, particularly in primary fibroblasts [202] (Figure 3). The exact implications of this interaction in the context of vascular remodeling and CVD warrant further investigation. Peripheral arterial disease (PAD), characterized by progressive atherosclerosis affecting the arteries of the lower limbs, poses significant health risks [203, 204]. Hakansson et al. [205] detected the levels of a specific set of snoRNAs at the 14q32 locus in the plasma of patients with end‐stage PAD and elite cyclists (Figure 3). Their findings revealed that SNORD114‐1 was highly expressed in patients with PAD, but its expression was low in elite cyclists. Interestingly, this study demonstrated for the first time that the level of SNORD114‐1 in the plasma was influenced by endurance exercise in elite cyclists. These findings suggest a potential association between SNORD114‐1 expression and PAD as well as the influence of endurance exercise on plasma levels. Further research is needed to elucidate the specific roles of snoRNAs, including SNORD114‐1, in vascular remodeling and PAD pathophysiology and their potential implications for CVD management and exercise physiology. The study by Nossent et al. [206] represents a continuation of the investigation of the role of snoRNAs, specifically SNORD112, SNORD113‐2, SNORD113‐6, and SNORD114‐1, in PAD. Their study demonstrated that these snoRNAs were highly expressed in the plasma of patients with PAD. Additionally, SNORD113‐2 and SNORD114‐1 levels were significantly negatively correlated with platelet activity, a key factor in the long‐term prognosis of PAD and CVD. These findings highlight the potential utility of SNORD113‐2 and SNORD114‐1 as prognostic indicators of PAD and CVD, although further studies are needed to confirm this hypothesis. In the context of hypertrophic cardiomyopathy (HCM), a common genetic heart disease, studies have revealed differences in snoRNA levels between wild type and HCM models involving extracellular vesicles (EVs) derived from human induced pluripotent stem cells (hiPSCs) differentiating into cardiomyocytes (hiPSC‐CMs) [207]. This suggests that snoRNAs may play a role in the pathophysiology of HCM; however, the specific mechanisms and implications require further investigation. Overall, these studies underscore the potential of snoRNAs as biomarkers and regulators of CVD such as PAD and HCM, indicating the importance of further research to elucidate their roles and clinical implications. The identification of snoRNAs in various studies, including those related to HCM, suggests their potential involvement in the pathogenesis of CVD [208, 209]. snoRNAs have also been implicated in conditions such as PAD, further highlighting their relevance to cardiovascular health. In the context of HCM, research by Tallo et al. [209] using a *Drosophila* model demonstrated differential expression of snoRNAs in response to genetic mutations associated with the disease. This suggests that snoRNAs play a role in HCM pathophysiology and could potentially serve as biomarkers for this condition (Figure 3). Early detection of CVD is crucial for effective clinical management. As traditional biomarkers may have limitations in terms of sensitivity and specificity, there is a growing interest in exploring novel biomarkers, such as snoRNAs. snoRNAs offer several advantages as potential biomarkers, including their stability and detectability in bodily fluids such as plasma or serum. Given the complexity and heterogeneity of CVD, further research is warranted to investigate the utility of snoRNAs as biomarkers in clinical practice. This includes large‐scale studies to validate diagnostic and prognostic values across different cardiovascular conditions. Ultimately, the integration of snoRNA‐based biomarkers into clinical practice could enhance early detection, risk stratification, and personalized treatment strategies for patients with CVD.

### 5.4 snoRNAs in Immunity and Inflammation

The study conducted by Lai et al. [210] provides valuable insights into the role of small nucleolar RNA 12 (SNORA12) in the pathogenesis of systemic lupus erythematosus (SLE), an autoimmune disease characterized by immune system dysfunction and tissue damage. Through transcriptome analysis of T cells from patients with SLE and healthy individuals, Lai et al. [210] identified differential expression of 18 ncRNAs, including SNORA12. Further investigation revealed that the expression of SNORA12 was significantly reduced in T cells from patients with SLE compared with that in healthy controls. This study elucidated the potential mechanism by which SNORA12 contributes to the immune pathogenesis of SLE. Overexpression of SNORA12 led to changes in the expression of CD69, a marker of T cell activation, and decreased expression of histone H4 lysine 20 (hist1h4k), which in turn inhibited the secretion of interferon‐gamma (IFN‐g), a key cytokine involved in immune responses [210]. These findings suggest that SNORA12 may modulate immune responses in SLE through its regulatory effects on CD69 and hist1h4k expression, ultimately impacting IFN‐g secretion. Understanding the role of SNORA12 in SLE pathogenesis may offer new insights into disease mechanisms and potential therapeutic targets. Further research is required to elucidate the precise mechanisms underlying SNORA12‐mediated immune dysregulation in SLE and its implications for disease management.

Research conducted by Fabien et al. [211] has shed light on the potential role of small nucleolar RNA 31 (SNORA31) in mediating the innate immunity of CNS neurons to herpes simplex virus type 1 (HSV‐1), specifically in the context of herpes simplex virus encephalitis (HSE). Their study involved five unrelated patients with HSE, each carrying a heterozygous mutation in SNORA31. Further investigation using cortical neurons derived from human pluripotent stem cells revealed that the knockdown of SNORA31 resulted in an increased susceptibility of these neurons to HSV‐1 infection. These findings suggest a potential role for SNORA31 in modulating the innate immune response of CNS neurons to HSV‐1 infection [211]. However, the specific mechanism by which SNORA31 influences neuronal immunity against HSV‐1 remains unclear and requires further investigation. Understanding the molecular mechanisms underlying the interaction between SNORA31 and HSV‐1 in CNS neurons could offer insights into the pathogenesis of HSE and potentially identify new therapeutic targets for the treatment of this serious neurological condition. Thus, further research in this area is warranted to fully comprehend the role of SNORA31 in CNS immunity and its implications in HSE.

The study conducted by Mandy et al. [212] provided valuable insights into the potential utility of snoRNAs as biomarkers for the diagnosis of OA, a degenerative joint disease characterized by the progressive destruction of articular cartilage and bone. They identified six differentially expressed snoRNAs (SNORD113, SNORA3, SNORD88, SNORA73, and SNORD38) in young and aging joints, indicating their potential involvement in joint aging. Subsequent animal experiments revealed that two snoRNAs, SNORD88 and SNORD38, exhibit significant changes during joint aging [212]. These findings suggest that snoRNAs, particularly SNORD88 and SNORD38, may serve as potential biomarkers for OA diagnosis. Differential expression of these snoRNAs in aging joints highlights their potential relevance to OA pathogenesis and progression. The identification of snoRNAs associated with OA could facilitate the development of noninvasive diagnostic tools for early detection and monitoring of OA progression. Further research is needed to elucidate the specific roles of snoRNAs in OA pathophysiology and their clinical utility as biomarkers for OA diagnosis and prognosis. The research findings of Mandy et al. [212] and Ellen et al. [214] have elucidated the involvement of specific snoRNAs in the pathogenesis of OA, particularly in the context of articular cartilage aging and chondrocyte differentiation. Mandy et al. identified associations between the expression of certain snoRNAs (SNORD96a, SNORD44, SNORD26, and SNORD116) and articular cartilage aging, which were also associated with articular cartilage OA [213]. This suggests that these snoRNAs may play a role in the molecular processes underlying age‐related changes in articular cartilage and the development of OA. Furthermore, Ellen et al. [214] demonstrated that the expression of SNORD3A is influenced by OA synovial fluid, and that bone morphogenetic protein 7 alters the phenotype of chondrocytes by modulating the expression of SNORD3A. They found that changes in SNORD3A expression affected chondrocyte differentiation as well as the regulation of rRNA levels and protein translation efficiency. Specifically, decreased SNORD3A expression is associated with reduced rRNA levels and translation ability, whereas increased SNORD3A expression has the opposite effect [214]. These findings highlight the multifaceted roles of snoRNAs in OA pathogenesis, including their involvement in articular cartilage aging, chondrocyte differentiation, and regulation of molecular processes essential for cartilage homeostasis. Understanding the mechanisms by which snoRNAs contribute to OA development and progression may offer new insights into the disease pathophysiology and potential therapeutic targets for intervention. Further research is necessary to fully elucidate the complex roles of snoRNAs in OA and their clinical implications.

A study involving 29 patients with COVID‐19 indicated a potential association between snoRNA expression and degree of inflammation in patients with COVID‐19. Specifically, significant differences in snoRNA expression were observed among patients with varying degrees of COVID‐19 severity, including severe, moderate, and asymptomatic cases, suggesting that the expression levels of the five snoRNAs were notably higher in patients with severe and moderate symptoms than in asymptomatic patients, indicating a potential correlation between snoRNA expression and the severity of COVID‐19 inflammation [215]. However, it is important to note that this study represents only one piece of evidence, and further research is needed to validate these findings and determine whether snoRNAs can serve as reliable biomarkers for predicting the degree of COVID‐19 inflammation. Additional studies with larger sample sizes and comprehensive analyses are necessary to confirm the utility of snoRNAs for evaluating COVID‐19 severity and inflammation. Nevertheless, these initial findings provide valuable insights into the potential role of snoRNAs in COVID‐19 pathogenesis, highlighting the importance of further investigations in this area.

These findings also underscore the intricate relationship between snoRNAs and macrophage polarization, which is crucial for the immune response and inflammation resolution. Macrophages play pivotal roles in the initiation and resolution of inflammation in response to various stimuli and pathogens. Studies suggest that snoRNA expression undergoes significant changes during macrophage polarization, indicating that snoRNAs may serve as regulators of this process. Macrophage polarization refers to the differentiation of macrophages into distinct functional phenotypes in response to microenvironmental cues such as proinflammatory or anti‐inflammatory signals. It has been hypothesized that the polarization state of macrophages can be altered by modulating the expression of snoRNAs, which influences their functional characteristics and immune responses [216]. This suggests a potential role for snoRNAs in fine‐tuning immune responses by regulating macrophage polarization. Further research is required to elucidate the specific snoRNAs involved in macrophage polarization and their mechanisms of action. Understanding how snoRNAs contribute to macrophage function and immune regulation may have implications for the development of therapeutic strategies targeting inflammatory and immune‐related disorders.

### 5.5 snoRNAs in Aging

Cellular senescence was first described by Hayflick in 1961. It occurs when cells reach a certain number of divisions, leading to “irreversible” arrest of their lifecycle. The expression of specific snoRNAs changes during cartilage aging and OA, and these changes are specifically associated with cartilage aging and OA. Moreover, the knockdown or overexpression of SNORD26 or SNORD96A has been shown to affect chondrogenesis, hypertrophy, rRNA expression, and the expression of OA‐related genes, indicating that SNORD26 and SNORD96A play crucial roles in these processes [213]. Research on osteoporosis in the elderly has shown that 63 snoRNAs undergo significant changes during the aging of bone marrow mesenchymal stem cells [217]. B cells are key components of the human immune system, and their aging is characterized by the gradual degradation of biological processes, leading to impaired immune function. This is accompanied by inflammaging, which is characterized by chronic low‐grade inflammation and a diminished ability to respond appropriately to immune stressors. As a result, infections are poorly controlled, which increases the risk of severe illness and even death [218, 219, 220]. Elderly individuals have significant B cell deficiencies, making them more susceptible to infections such as COVID‐19 and more prone to stronger adverse reactions to vaccinations such as the flu vaccine [221, 222, 223]. Further research has revealed that in aged mice, the expression of SNORD123 and Cdkn2a is increased in B cells compared with younger mice. This suggests that B cell senescence may contribute to the weakened immune response observed with aging. The varying expression levels of SNORD123 across different age groups indicates that it may play a crucial role in this process, warranting further investigation [224]. Cellular senescence is an irreversible state of cell cycle arrest that is triggered by various stressors, including abnormal oncogene activation, telomere shortening, and DNA damage. Researchers identified a conserved snoRNA, SNORA13, which is crucial for various forms of senescence in both human cells and mice. During HRASG12V‐induced senescence in human fibroblasts, SNORA13 was found to directly interact with RPL23, slowing the assembly rate of the mature 60S ribosomal subunit and negatively regulating ribosome biogenesis. This leads to the accumulation of free ribosomal proteins, which in turn trigger p53 activation [225]. These findings reveal a novel mechanism of snoRNAs in cellular signaling, deepening our understanding of the molecular mechanisms underlying cellular senescence and paving the way for further extensive research into the diverse roles of snoRNAs (Table 1).

**TABLE 1:** Summary of snoRNA‐associated diseases and their mechanisms.

Disease	SnoRNA/target	Mechanism	References
LCC	SNORD118/small nucleolar RNA U8	Affects the 3′ end processing of precursor U8.	[168]
MDD and SCZ	SnoRNAs, such as hbi‐52 and hbi‐36/serotonin 5‐HT2C receptor SNORA69/5.8S rRNA or 18S rRNA	Regulation of 5‐HT2C mRNA expression. Affects the binding ability of rRNA with other molecules, such as tRNA and mRNA, thereby impacting the translation process and the proteomic profile within the cell.	[178, 180]
AS	SNORD113‐6/tRNAs	snoRNAs are involved in processing/splicing pre‐mRNA and 2′‐O methylation of mRNA, ensuring the stability of mRNA expression.	[206]
SLE	SNORA12/CD69 and histhh4k	Regulation expression of hist1h4k, which in turn inhibited the secretion of IFN‐g.	[210]
OA	SNORD3A/OA synovial fluid	Regulation of rRNA levels and protein translation efficiency.	[214]
Senescence	SNORA13/RPL23	SNORA13 was found to directly interact with RPL23, slowing the assembly rate of the mature 60S ribosomal subunit and negatively regulating ribosome biogenesis. This leads to the accumulation of free RPs, which in turn triggers p53 activation.	[225]

Abbreviations: 5‐HT2C, 5‐hydroxytryptamine receptor 2C; AS, arterial atherosclerosis; hist1h4k, histone H4 lysine 20; IFN‐g, interferon‐gamma; LCC, leukoencephalopathy with calcifications and cysts; MDD, major depressive disorder; OA, osteoarthritis; RPs, ribosomal proteins; SCZ, schizophrenia; SLE, systemic lupus erythematosus.

## 6 Recent Technological Advances in snoRNA Research

### 6.1 High‐Throughput Sequencing

Given the critical biological functions of snoRNAs in both physiological and pathological processes, advancements in technologies for detecting their expression are of significant scientific and clinical importance. As mentioned previously, snoRNAs are typically 60–300 nt long, making them smaller than many other types of RNAs. This size can complicate detection, particularly with methods like northern blotting or RNA‐seq, where RNA length affects detection efficiency. snoRNAs differ from other RNAs in the following ways regarding detection techniques: (1) Northern blotting: This traditional method is commonly used for detecting snoRNAs, but because of their small size, specific probes and optimized conditions are required [226, 227, 228]. (2) Quantitative reverse transcription PCR (qRT‐PCR): While widely used for other RNAs, such as mRNAs and miRNAs, qRT‐PCR for snoRNAs requires specific primers that can amplify these small sequences accurately. It often necessitates the use of specialized reverse transcription protocols to ensure efficient cDNA synthesis from snoRNAs [229]. (3) RNA sequencing (RNA‐seq): Although RNA‐seq is used for various RNA types, snoRNAs often require specific library preparation techniques to enrich for small RNAs or to avoid being overlooked in favor of more abundant RNA species. Specialized bioinformatic tools are required to accurately map and quantify snoRNAs [230, 231]. (4) In situ hybridization: This technique visualizes snoRNAs in their nucleolar localization, differing from mRNAs, which are found in various cellular compartments [232, 233, 234, 235]. As snoRNAs play a crucial role in regulating RNA modifications such as 2′‐O‐methylation and pseudouridylation, which are essential for RNA stability, processing, and function. The development of high‐throughput sequencing technologies, holds great promise for advancing our understanding of snoRNA functions and mechanisms. For example, high‐throughput detection of RNA 2′‐O‐methylation can be achieved using chemical modification sequencing techniques, such as RiboMethSeq [236] dynamic RNA modifications in gene expression regulation, while RNA pseudouridylation can be detected through chemical labeling and high‐throughput sequencing methods, such as Pseudo‐seq [237]. Recently, the single‐molecule imaging technique has been successfully employed to visualize each step of miRNA‐mediated gene silencing in situ within cells [238]. This technique holds significant potential for detecting snoRNAs and visualizing their interacting RNAs. To further elucidate the interacted RNAs of snoRNAs, Liu and et al [239, 240]. developed a snoRNA‐enriched kethoxal‐assisted RNA‐RNA sequencing (snoKARR‐seq) to mapping snoRNA targets transcriptome‐wide. This approach uncovered noncanonical functions for snoRNAs in protein translocation and secretion.

These technological advances have not only expanded the understanding the diverse biological functions of snoRNAs, and their association with numerous diseases, but also provided potential diagnostic and therapeutic tools.

### 6.2 Structural Biology

With the development of new technologies, the study of snoRNA structures has become increasingly advanced. Among these technologies, cryo‐electron microscopy (cryo‐EM) has emerged as a crucial tool for investigating the three‐dimensional structures of RNA and RNA‐protein complexes. It provides high‐resolution structural information, which significantly enhances our understanding of snoRNPs. A study has used cryo‐EM to deepen the understanding of mutations associated with telomerase‐related diseases and the pseudouridylation mechanism of eukaryotic H/ACA RNPs [84]. Single‐molecule imaging techniques can be used to directly observe the structural changes of RNA molecules. By labeling snoRNA, its spatial structural variations can be tracked [241]. The development of computational biology tools has accelerated the study of snoRNA structures. For example, computational tools like RNAfold, Mfold, and SHAPE‐seq can be used to predict the secondary structure of snoRNAs. Among them, SHAPE‐seq allows for RNA structure prediction at the transcriptional level [242, 243, 244]. Research on snoRNA structures is enhancing our understanding of its functions and mechanisms. While snoRNAs have been found to play roles in various diseases, the exact mechanisms of action remain unclear. These advanced techniques will help deepen our understanding of how snoRNAs contribute to disease processes.

### 6.3 Bioinformatics Tools

Over the past two decades, advances in cutting‐edge technology have transformed the research landscape, enabling a deeper understanding of the biology, localization, and functions of snoRNAs [245]. snoRNAs have several distinct characteristics that differentiate them from other types of RNA. The primary characteristics of snoRNAs are reflected in the following five areas: function, location, structure, transcription, processing, and regulation. The snoDB database integrates information from diverse sources of human snoRNAs, including the latest data on snoRNA characteristics, genomic locations, host genes, snoRNA–RNA targets, and snoRNA abundance [246]. The second edition, snoDB 2.0, includes sections on rRNA chemical modifications, snoRNA motifs, secondary structure predictions, snoRNA–protein interactions, and low‐structure‐bias expression data across a wide range of tissues and cell lines. This database is instrumental for investigating snoRNA biology [247]. The sRNAfrag database provides a comprehensive foundation for quantifying and analyzing small RNA fragments and advancing small RNA research [248]. For studies of snoRNAs in diseases, exploring snoRNA functions and identifying their binding sites are crucial. The snoGloBe database, a human C/D snoRNA interaction predictor based on a gradient‐boosted classification, is useful in this context. snoGloBe considers interaction target type, location, and sequence. Specific snoRNAs can identify interactions near regulatory elements of gene expression, including splice sites, thereby predicting target abundance and related splicing changes. Researchers can use snoGloBe to identify experimentally validated binding sites and predict new sites with shared regulatory functions [249]. Additionally, the iSnoDi‐MDRF database, which links snoRNAs with diseases based on multiple biological datasets, can be used to combine known snoRNA–disease associations. This enables the training of effective models, and through machine learning, the exploration of potential snoRNA–disease mechanistic relationships [250, 251]. RESTART, a programmable RNA base editor, could also be used. This editor relies on guide snoRNAs to achieve the pseudouridylation of primary cells. It effectively induces pseudouridylation at nonsense mutation sites in disease‐related contexts, thereby improving protein function. RESTART is used for RNA editing in the study and treatment of various diseases [76]. Recent studies have utilized a tool called sRNAfrag that can quantify and analyze small RNA fragments across various organisms. Based solely on sequencing data, this tool can identify miRNA cleavage sites [252], providing a functional foundation for further research on small RNAs. Another study validated the expression of 106 C/D box snoRNAs, of which 31 were predicted to target novel 2′‐hydroxyl ribose sites in rRNA. This lays the groundwork for understanding the effect of snoRNA‐mediated 2'‐O‐methylation on translation and protein stability in organisms [253]. Rfam is a comprehensive database of ncRNA families, where each family is characterized by multiple sequence alignments, consensus secondary structures, and covariance models. By converting descriptions of RNA families from the literature into computational models, Rfam enables the annotation of RNA or DNA sequences that belong to these families. The research findings, including charts and supplementary information files, are accessible through the Rfam website. The data generated by Rfam are widely utilized, ranging from genome annotation to providing training sets for algorithm development [254]. On the other hand, the snOPY database offers comprehensive information on snoRNA gene loci and their target RNAs. It also includes data on orthologs from various species, facilitating the analysis of snoRNA gene evolution [255]. Together, these resources provide valuable tools for studying the structure, function, and evolutionary dynamics of ncRNAs (Table 2).

**TABLE 2:** Technological advancements in snoRNA research and their applications.

Technological	Application	References
High‐throughput sequencing	High‐throughput identification of C/D box snoRNA targets	[256]
Cutting‐edge RNA‐sequencing technology	Cutting‐edge RNA‐sequencing technology to identify biomarkers and potential therapeutic targets	[257, 258]
Drug delivery nanoparticles	LNP can deliver RNA (both siRNA and mRNA) to HSCs in vivo	
snoDB database	The snoDB database including the latest data on snoRNA characteristics, genomic locations, host genes, snoRNA–RNA targets, and snoRNA abundance	[246]
sRNAfrag database	The sRNAfrag database provides a comprehensive foundation for quantifying and analyzing small RNA fragments and advancing small RNA research.	[248]
snoGloBe database	The snoGloBe database considers interaction target type, location, and sequence.	[249]
iSnoDi‐MDRF database	The iSnoDi‐MDRF database can be used to combine known snoRNA–disease associations.	[250]
RESTART	A programmable RNA base editor	[76]
Rfam	Rfam is a comprehensive database of noncoding RNA families, where each family is characterized by multiple sequence alignments, consensus secondary structures, and covariance models.	[254]
snOPY database	The snOPY database offers comprehensive information on snoRNA gene loci and their target RNAs.	[255]

Abbreviation: LNP, lipid nanoparticle.

## 7 Challenges and Future Directions

### 7.1 Biological Complexity of snoRNAs

The study of snoRNAs has become increasingly interdisciplinary, with researchers from various fields contributing to our understanding of these molecules. The cross‐collaboration between snoRNA research and other disciplines has led to significant advances in the field. Molecular biologists have contributed to understanding the structure and function of snoRNA, including its role in ribosome biogenesis and RNA modification mechanisms. Geneticists have contributed to identifying and characterizing snoRNA genes, including their genomic organization and expression patterns. The study of snoRNA mutations has provided insights into their functional importance and the consequences of dysregulation in disease. Clinicians have identified links between dysregulation of snoRNAs and various diseases, such as cancer, neurological disorders, and developmental abnormalities. Researchers have also investigated the potential use of snoRNAs as biomarkers for disease diagnosis and prognosis. Computational biologists have developed bioinformatics tools to facilitate the study of snoRNAs by enabling their identification, prediction, and analysis. Additionally, computational models have been utilized to investigate the intricate interactions between snoRNAs and other cellular components, providing valuable insights into their regulatory roles. snoRNAs have been found to regulate DNA damage through noncanonical pathways. Specifically, SNORA73 influences DNA damage by forming an snoRNP complex with PARP1 and the canonical H/ACA proteins DKC1/NHP2. This interaction affects genomic stability, thereby playing a role in gene regulation [98]. snoRNAs may also interact with other RNAs, such as mRNAs. However, due to the lack of effective techniques for transcriptome‐wide identification of snoRNA targets, a study developed a chemical crosslinking‐based method to comprehensively detect cellular RNA targets of snoRNAs. This approach revealed previously unidentified snoRNA–mRNA interactions in human cells and mouse brain tissues. Notably, these interactions occurred outside the sites of snoRNA‐guided RNA modifications. The study also identified that SNORA73 targets mRNAs encoding secretory and membrane proteins. Furthermore, SNORA73 interacts with 7SL RNA, and the mRNA–SNORA73–7SL RNA interaction promotes the secretion of encoded proteins. This discovery highlights a novel function of snoRNAs that extends beyond their previously known roles [239]. More importantly, snoRNAs can be released into EVs, and abnormal expression of snoRNAs can be detected in the blood of patients with certain diseases. For example, differential snoRNA expression has been observed in the early stages of AD, aiding in early diagnosis and treatment, which can significantly improve patient outcomes [256]. Similarly, snoRNAs have been detected in EVs in the plasma of patients with BC, HCM, and following immune activation or inhibition of dendritic cells [208, 257, 258]. Collectively, these findings suggest that snoRNAs are likely to emerge as therapeutic targets for diseases in the future. Nevertheless, further studies are required. These findings highlight the biological complexity of snoRNAs. Precisely because of this complexity, collaborative efforts from experts across various fields are essential. Thus, the combination of expertise from various fields has resulted in a more comprehensive understanding of snoRNA biology. Collaborations across disciplines have led to the development of innovative tools and methods for researching snoRNAs. The integration of knowledge from diverse fields may unveil new areas of investigation, such as exploring the role of snoRNAs in diseases and their potential as therapeutic targets. Future developments may involve the integration of artificial intelligence to elucidate and predict the mechanisms and target points of snoRNAs in diseases. Identifying snoRNAs as common therapeutic targets could help develop optimal treatment strategies, marking a new direction in this field. Comprehensive advancements, from technological methodologies to structural and functional research, are required to further elucidate their biological intricacies and translate these insights into clinical applications. These technologies include the development of reliable delivery systems, optimized dosing regimens, and strategies to mitigate off‐target effects of siRNAs. Once these technical limitations are overcome, snoRNAs, with their specific regulatory functions, hold significant potential as therapeutic targets.

### 7.2 Translation to Clinical Applications

So far, studies have revealed snoRNAs in liver cancer. SNORD17, SNORD53, snoRNA ACA11, snoRNA U2‐19, SNORA23, SNORA11, SNORD124, SNORD46, SNORD88B and SNORD52 in liver cancer and suggest their potential as therapeutic targets or biomarkers [115, 116, 117, 118, 119, 120, 121, 122, 123]. In lung cancer, SNORA80E, and SNORD71A providing potential targets for therapeutic interventions [125, 126]. SNORA51 may serve as an important biomarker and potential therapeutic target, offering new insights into the diagnosis and treatment of BC and potentially providing valuable implications for research on other malignant tumors [133]. SNORA24 and SNORA28 have potential value as a biomarker and therapeutic target for patients with CRC [139, 140, 141, 142]. Studies on gastric tumors have suggested SNORA42, SNORA74A, and SNORD10 require further investigation to ascertain their potential as therapeutic targets [145]. SNORD49A, SNORD55, SNORD105, SNORD34, SNORD35A, SNORD43, SNORD104, SNORD118, and SNORD110 provide valuable insights into the potential roles of snoRNAs in leukemia pathogenesis [146, 147]. In MDSs, SNORD115 and SNORD116 provide valuable insights into disease mechanisms and potential therapeutic targets [171]. SnoRNAs, such as SNORD118, E307 and E470 suggest their potential as early diagnostic markers of LCC [168, 170]. SNORD115, SNORD116, and SNORA69 may be a potential biomarker for predicting MDD and SCZ [179]. SNORD115 and SNORD116 offer a valuable tool for studying the neurological aspects of PWS and potentially for developing therapeutic interventions to address cognitive impairments associated with the syndrome [185, 186].

Research on snoRNAs in various diseases has highlighted their significant potential for clinical applications, particularly as biomarkers for disease diagnosis and as therapeutic targets. However, clinical trials have not confirmed these findings. In our search of clinical trial databases, we identified a preclinical study related to snoRNAs. This study primarily examined the diagnostic and prognostic value of circulating exosomal small RNAs (including miRNA, snoRNA, tRNA, and piRNA) in patients with pancreatic cancer. The study involved 68 patients with pancreatic cancer and a control group of 34 patients with other pancreatic conditions, including MCN, SCN, IPMN, SPN without malignant pathology, chronic pancreatitis, and cholangiocarcinoma. In the study, 12 mL of venous blood was collected from each participant to assess the sensitivity and specificity of the exo‐sRNAs in the blood of patients with pancreatic cancer. The findings of the study revealed that these exosomal sRNAs demonstrated greater specificity than CA19‐9, a United States Food and Drug Administration‐approved biomarker for pancreatic cancer diagnosis. In addition, exosomes protect RNA from degradation by plasma RNases. Small RNAs, including miRNAs, snoRNAs, tRNA, and piRNAs, are more stable than long RNAs, highlighting their untapped potential [259]. This presents a promising research direction for researchers interested in exploring small RNA applications.

Emerging preclinical studies have demonstrated the potential of snoRNAs as promising biomarkers and therapeutic targets across diverse pathologies. In cancer research, snoRNAs are being explored for validating treatment efficacy in pancreatic cancer (NCT04636788) and as biomarkers for estrogen dependency in Luminal‐B breast cancer (NCT06805084). Additionally, the potential of specific snoRNA SNORD3A as a novel circulating biomarker in ischemic heart disease and heart failure is currently under investigation [260, 261, 262] (NCT06678802). Notably, research on snoRNAs in PWS has revealed critical insights: SNORD116‐deficient PWS mouse models with paternal inheritance exhibit significant delays in processing short‐term temporal intervals (ranging from milliseconds to minutes), leading to disrupted feeding behaviors and altered REM sleep architecture. These deficits ultimately impair the organization of REM episodes across sleep‐wake cycles [263, 264].

In parallel, preclinical studies have identified the tumor suppressor gene GAS5, which encodes both lncRNAs and snoRNAs. Elevated expression of GAS5‐derived transcripts postchemotherapy correlates with enhanced apoptosis and reduced survival in breast cancer cells, highlighting its therapeutic implications [265]. While current preclinical evidence remains limited, these findings collectively underscore the translational potential of snoRNAs in clinical practice. Their dual utility—as diagnostic biomarkers and therapeutic agents—could substantially refine existing treatment paradigms for multiple diseases.

### 7.3 Emerging Frontiers

**FIGURE 4:**
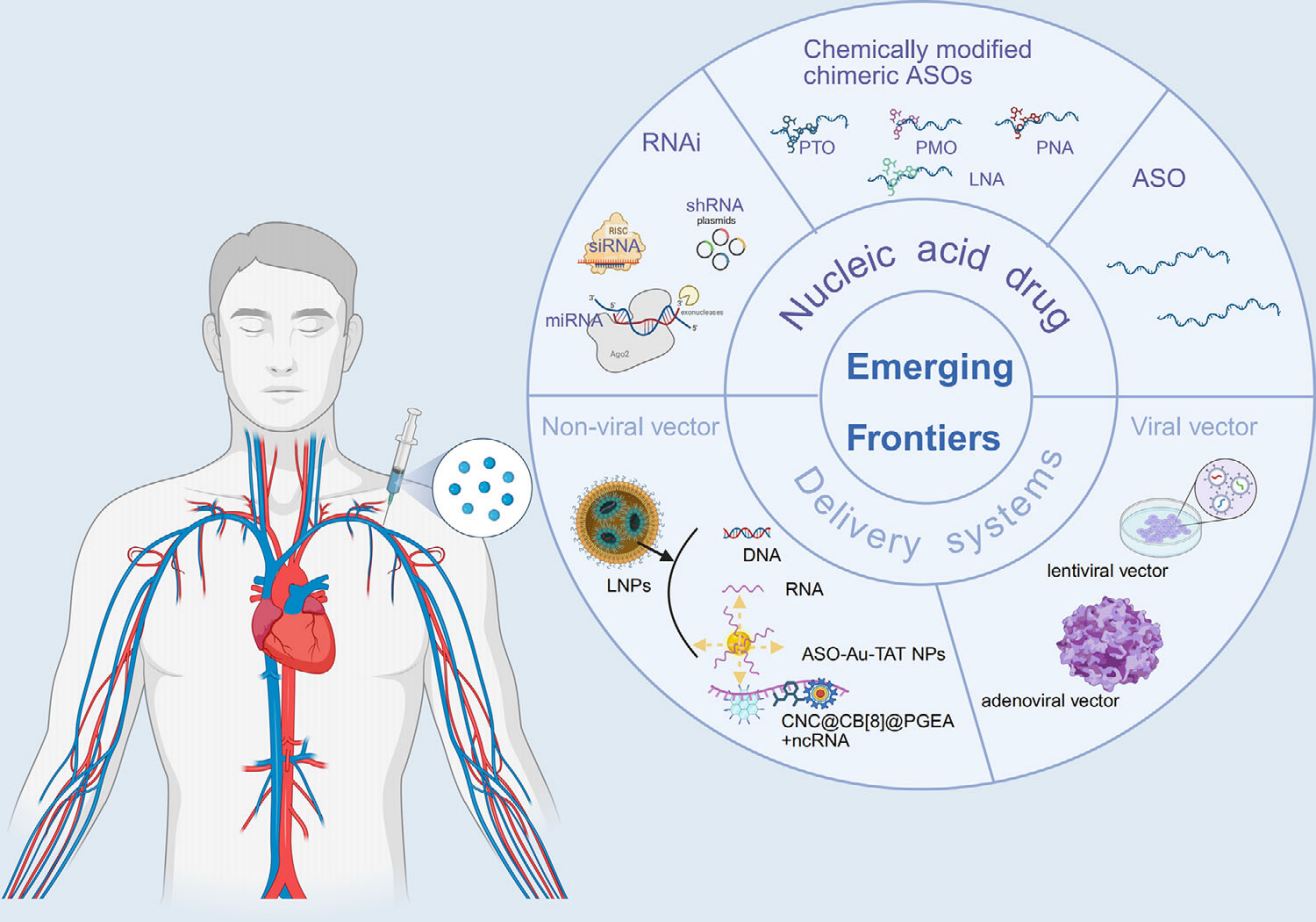
Emerging frontiers in the snoRNA filed include the development of nucleic acid drugs targeting snoRNAs, as well as the improvement of delivery systems for snoRNAs and ASOs. Abbreviations: RNAi, RNA interference; ASO, antisense oligonucleotide; LNPs, lipid nanoparticles; PTO, phosphothioate oligonucleotides; PMO:phosphorodiamidate morpholino oligomer; PNA, peptide nucleic acid; LNA, locked nucleic acid; CNC@CB[8]@PGEA, a novel rodlike supramolecular nanoassemblies, consisting of the unique properties of cellulose nanocrystals (CNCs), polycations (ethanolamine‐functionalized poly (glycidyl methacrylate) (PGEA), and poly (aspartic acid) (PAsp). Figure 4 was created with BioRender.

Numerous snoRNAs have been identified across various organisms, playing significant roles in regulating gene expression and contributing to human diseases [266]. Recent large‐scale transcriptomic analyses have revealed that the majority of snoRNAs are localized in the nucleus. While RNA interference (RNAi) is commonly used to analyze protein‐coding genes and effectively depletes cytoplasmic mRNAs, it is less efficient at targeting nuclear RNAs [267]. Conventional transfection of chemically modified ASOs is an effective method for depleting snoRNAs in human and mouse cells. These oligonucleotides facilitate RNaseH‐mediated cleavage of target RNAs. ASO targeting is highly specific, as it does not affect the expression of host genes harboring snoRNA‐embedded introns or alter the levels of snoRNA isoforms with high sequence similarity. Notably, at least five snoRNAs can be simultaneously depleted using this approach [266]. Current studies have demonstrated that ASO‐mediated silencing of SNORD89 can inhibit the proliferation and migration capabilities of endometrial cancer cells [268]. Additionally, intratumoral injection of ASO–SNORD6 resulted in slowed tumor growth and reduced tumor volume [269]. Subsequent studies have refined the system for knocking down snoRNAs using chemically modified ASOs. These numerous chemical modifications, including phosphothioate oligonucleotides, phosphorodiamidate morpholino oligomer, peptide nucleic acid, and locked nucleic acid, enhance ASOs resistance to nuclease, prolong tissue half‐life and reduce nonsequence‐specific toxicity [270]. For example, the phosphorothioate‐modified ASOs enable efficient and specific degradation of at least 20 different nuclear ncRNAs including snoRNAs across various mammalian cell lines [267]. In summary, a convenient and effective method to modulate nuclear ncRNAs in mammalian cells, along with the development of antisense drugs targeting disease‐associated ncRNAs, serves as a powerful tool for exploring the biological functions of the vast number of nuclear ncRNAs with unknown roles (Figure 4).

Delivery systems are crucial for realizing the therapeutic potential of snoRNA‐based drugs. An ideal delivery system must exhibit satisfactory specificity, stability, cell permeability, and low immunogenicity. Classical delivery vehicles are primarily divided into viral and nonviral vectors. Viral vectors, including lentiviral and adenoviral vectors, have been largely replaced by other delivery systems due to concerns over their safety [271, 272]. Among nonviral vectors, liposomes are ideal materials for the targeted delivery of snoRNAs, especially considering advancements in nanotechnology. Nanoliposomes hold significant potential for both functional studies and therapeutic applications of snoRNAs [272]. Liposome encapsulation not only reduces toxicity and immunogenicity but also enhances drug stability and liver tissue targeting. It ensures that the encapsulated material is not filtered out by the kidneys and is gradually absorbed by target cells in the liver during systemic circulation. Several liposome‐encapsulated DNA (NCT01502358) or mRNA (NCT03382405) delivery systems have advanced to clinical trials. Additionally, liposomes are widely used for ncRNA delivery in both in vitro and in vivo preclinical studies [273, 274, 275]. Lipid nanoparticles (LNPs) are similar to liposomes but offer greater diversity and are more suitable for encapsulating a variety of nucleic acid‐based drugs. As a result, LNPs have become the most widely used nonviral delivery system in gene therapy [276, 277]. Recently, in a xenograft lung cancer mouse model, the application of ASO–Au–TAT nanoparticles (NPs) targeting the nuclear lncRNA MALAT1 significantly inhibited tumor metastasis and extended the survival time of mice by 80%. This remarkable outcome is attributed to the NPs' highly specific nuclear localization capability for ASOs [278]. These findings demonstrate that NPs conjugated with cell‐penetrating peptides (such as TAT) can enhance the application of ASOs for targeting nuclear ncRNAs. Furthermore, novel rodlike supramolecular nanoassemblies (CNC@CB[8]@PGEA) consisting of the unique properties of cellulose nanocrystals (CNCs), polycations (ethanolamine‐functionalized poly(glycidyl methacrylate) (PGEA), and poly(aspartic acid) (PAsp), can condense into nanocomposites with particle sizes smaller than 200 nm when cotransfected with MEG3 and miR101. This enables the synergistic delivery of ncRNAs of varying sizes, enhancing drug targeting and therapeutic efficacy [279]. These advancements suggest that, with the continued development of such technologies, the delivery of snoRNAs as nucleic acid‐based drugs in vivo will soon become a reality (Figure 4).

NP‐based drugs have been approved for the treatment of human cancers [280]. The research and development of drug delivery NPs is ongoing, with articles summarizing more intelligent and efficient delivery strategies for NPs in drug delivery [281]. Additionally, one study has provided a comprehensive approach for evaluating RNA‐based LNPs, offering guidance for researchers in the field of snoRNAs and LNPs for disease treatment [282]. Although current research on snoRNAs and tumor therapy remains largely speculative, snoRNAs have demonstrated value in tumor diagnosis and prognosis [283]. Continued exploration of snoRNA involvement in tumorigenesis may lead to the development of novel therapeutic strategies (Figure 4).

RBPs are a crucial class of proteins in cells that interact with RNA to perform essential functions, playing a vital role in all eukaryotic cells [284]. RBPs can also interact with snoRNAs. For instance, studies have shown that the C/D box protein L7Ae plays a role in ribosome biogenesis as well as in the biogenesis of H/ACA and C/D box snoRNPs by binding to K‐turn motifs [285]. Additionally, nucleolin, one of the major RBPs in the nucleolus, is involved in the early cleavage of pre‐rRNA. Nucleolin interacts with the U3 snoRNP, activating the processing activity of S100 cell extracts and playing a role in primary processing reactions and ribosome biogenesis [286]. RBPs can recognize snoRNA‐binding domains and interact with them, potentially enabling their widespread involvement in various posttranscriptional regulatory processes, including RNA splicing, transport, sequence editing, intracellular localization, and translation control. Research on the interactions between RBPs and snoRNAs represents a critical area for future breakthroughs in this field.

## Author Contributions

Y.W.: writing—original draft. M.F.: visualization. Z.Z.: Visualization and software. J.F.: conception and design of the study. C.Z.: manuscript review and editing. All authors contributed to the manuscript and approved the submitted version.

## Ethics Statement

The authors have nothing to report.

## Conflicts of Interest

The authors declare no conflicts of interest.

## Data Availability

The authors have nothing to report.
